# Formulating a concept of operation (ConOps): A stakeholders-assisted standardization process for developing an integrated wildfire management platform

**DOI:** 10.1007/s13280-026-02375-1

**Published:** 2026-05-03

**Authors:** Yvonne Brodrechtova, Andrea Majlingová, Zoltán Balogh, Emil Gatial, Ján Zelenka, Maroš Sedliak

**Affiliations:** 1 Faculty of Forestry, Technical University in Zvolen, T. G. Masaryka 24, 96001 Zvolen, Slovakia; 2 Faculty of Wood Sciences and Technology, Technical University in Zvolen, T.G. Masaryka 24, 96001 Zvolen, Slovakia; 3 Institute of Informatics of the Slovak Academy of Sciences in Bratislava, Dúbravská cesta 9, 845 07 Bratislava, Slovakia

**Keywords:** Concept of operations (ConOps), Stakeholder participation, Systems engineering, Technological and information platform, Wildfire management

## Abstract

Wildfire management is undergoing rapid transformation as data fusion, artificial intelligence, and participatory approaches are used to enhance ecosystem resilience and community safety. This paper presents the development of a concept of operations (ConOps) within the EU project SILVANUS, aimed at supporting design of an integrated, data-driven platform for wildfire management. The ConOps was developed using a methodology that combines systems engineering principles, standardized information management, and participatory stakeholder engagement across ten international pilot sites. Through iterative refinement, it captures the operational context and system requirements in pre-fire, active fire, and post-fire phases. The analysis identifies key limitations in existing wildfire management systems such as fragmented technologies, inconsistent data standards, limited interoperability, and weak institutional coordination. The proposed ConOps provides a structured and replicable framework to address these challenges. While the methodology effectively supports requirement definition and operational scenario development, real-world pilot testing remains necessary to assess implementation challenges.

**Supplementary Information:**

The online version contains supplementary material available at https://doi.org/10.1007/s13280-026-02375-1.

## Introduction

There is high confidence in the global increase in both the intensity and frequency of wildfires (Bowman et al. 2009; Huang et al. 2015; Forzieri et al. 2016). This escalation is driven by multiple interrelated factors, including climatic variability (e.g., humidity, temperature, and wind), ecological conditions (e.g., fuel load), and socio-economic influences such as human activities, land use change, and the expansion of urban infrastructure. In response, significant technological and technical advances have been made in wildfire management, including improvements in firefighting equipment, communication tools, tactical response strategies, and analytical support capabilities, particularly in developed countries (Valero et al. 2017; Kamilaris et al. 2023). Although developing countries have begun to adopt these innovations, their implementation remains uneven due to challenges related to institutional capacity, infrastructure, and governance (De et al. 2019; Nunoo et al. 2024).

Despite these advances, information management remains overlooked amid the prevailing focus on operational activities. Yet, this rapidly evolving domain holds substantial potential to improve wildfire management. The exponential growth of data and the proliferation of computing platforms have resulted in fragmented and often inefficient data management within and across organizations. To mitigate this fragmentation, recent research has explored the potential of digital technologies such as remote sensing, virtual reality, the internet of things (IoT), artificial intelligence (AI), and advanced early warning and fire detection systems for wildfire prediction and monitoring (Boroujeni et al. 2024; O’Mara et al. 2024; Shaik et al. 2025; Zhai et al. 2025). Nevertheless, a significant research gap persists regarding technology-driven information systems and integrated digital platforms designed specifically to support wildfire management. Such platforms could enable essential data sharing, analytics, and coordination tools for stakeholders, facilitating seamless integration of operational capacities across all three phases of wildfire management: pre-fire (A), during-fire (B), and post-fire (C) (in sensu Huidobro et al. 2025).

Against this background, the European Union’s Horizon 2020 funded SILVANUS project^1^ focused on the integration and demonstration of innovative, user-oriented solutions primarily across European Union (EU) countries. The project aimed to develop an integrated technological and information system for wildfire management, providing near-real-time data on forestry, weather, and community status to support rapid and holistic decision-making. The platform architecture envisaged the integration of system modules supporting citizen engagement, wireless communications, biodiversity modeling, coordinated UAV/UGV operations, and fire behavior modeling. A persistent challenge in systems engineering is aligning developers’ concepts with stakeholder needs—particularly those of end users—a difficulty compounded by incomplete knowledge of operational environments and their constraints (Korfiatis et al. 2012; de Miguel-Díez and Purfürst, 2025). The development of such platforms requires extensive, standardized data that influence platform performance and depend on the active participation of stakeholders providing the data. Attention must be given to the standardization of data collection, data sharing, and data management.

In systems engineering practice, the concept of operations (ConOps) serves as a user-centered framework for the development of complex systems by articulating operational needs and system expectations from the user’s perspective (e.g. Faulconbridge and Ryan 2002; Korfiatis et al. 2012; Mostashari et al. 2012). A ConOps document outlines the characteristics and intended use of a proposed system, emphasizing how it will function within the operational environment. It typically describes the current operational state, plausible use-case scenarios, and desired future requirements for the system, and is therefore recommended for preparation at the early stages of the system engineering lifecycle (Jost 2007; Nelson 2007).

The ConOps framework has been applied in various domains, including the development of UAV swarm systems for wildfire management (Karvonen et al. 2023; Sadrabadi et al. 2025). However, while ConOps offers high-level guidance for aligning system design with user requirements, it provides limited detail on the techniques, processes, and methodologies required for its systematic formulation (Olayan et al. 2018). To support the development of ConOps documentation for wildfire management platform design, this study proposes a systematic methodology tailored to wildfire management systems that explicitly accounts for current operational environments, existing constraints, and system requirements. Central to the proposed methodology is structured information management, encompassing data collection, evaluation, and standardization. These are processes essential for producing actionable and evidence-based ConOps documentation.

The remainder of this paper is organized as follows: first, relevant ConOps literature is reviewed; second, a step-by-step methodology for ConOps development methodology in wildfire management systems is presented; and finally, results and discussion are provided based on methodology application across eight EU countries and Indonesia.

## Theoretical framework

### Technological platforms: Current systems, situations, and context

In the digital sector, technological platforms have become central to value creation, driven by key firms often described as platform leaders (Gawer and Cusumano 2002) or keystone firms (Iansiti and Levien 2004). Examples such as Google, Apple, and Facebook illustrate how dominant companies shape and control platform ecosystems through technological and strategic influence (Gawer 2014). Research on platforms has evolved along two lines (Dougherty and Dunne 2011): (i) an economic perspective, emphasizing platform competition, pricing, and market dynamics, and (ii) an engineering design perspective, focusing on platform innovation, modularity, and system architecture. Despite these different analytical lenses, a common element across organizational contexts is the primacy of platform design (Huang et al. 2005; Simpson et al. 2006; Jiao et al. 2007).

According to Baldwin and Clark (2000), a system’s architecture specifies the constituent components and their roles, whereas interfaces define how these components connect, align, and communicate. Numerous studies highlight that platforms typically rely on modular technological architectures (e.g., Gawer 2009, Baldwin and Woodard 2008), enabling flexibility and scalability (Ulrich 1995; Baldwin and Clark 2000). The main advantage of modular architecture lies in the clear specification of interfaces, which enables interoperability among modules, while allowing decentralized development and preserving overall system integrity (Baldwin and Clark 2000). In other words, in digital platforms like iOS or Android, developers build applications (modules) that interface with a core system, similarly, in product ecosystems modularity enables mass customization and scalable innovation.

### The role of ConOps in the development of complex systems

The fields of intelligent systems, real-time systems, and software engineering have expanded significantly in recent decades (Henao et al. 2001). The development of such platforms requires structured and standardized methodologies that integrate appropriate tools, techniques, and processes to ensure successful system delivery and quality. Ad hoc approaches such as those lacking formal planning, documentation, or stakeholder analysis are inadequate to address the complexity and reliability demands characteristic of these systems. A key instrument for defining the strategy to achieve a system’s mission is the ConOps document, introduced by Fairley and Thayer (1997). Initially developed for military and government applications, ConOps has since been widely adopted in commercial software engineering, particularly in the design and modification of software-intensive systems in which software constitutes the primary technical challenge. Such systems typically comprise integrated combination of hardware, software, people, and manual procedures. They may be described either functionally, focusing on purpose and success criteria, or physically, emphasizing system components, interactions, production processes, and integration strategies (Faulconbridge and Ryan 2002). For instance, a wildfire management system can be conceptualized as an integrated assembly of hardware, software, and human components working collaboratively toward common objectives (e.g., Sakellariou et al. 2017; Lourenço et al. 2021; Vásquez et al. 2021; Ghali and Akhloufi 2023).

The first formal standardization of ConOps appeared in the IEEE Guide for Information Technology—System Definition (IEEE 1362-1998) and was later reinforced by international standards such as ISO/IEC/IEEE 29148:2011, ANSI/AIAA G-043A:2012, and ISO/IEC/IEEE 15288:2015. This progression reflects a broader shift from traditional business planning approaches toward more detailed and operationally oriented system design frameworks. Within this context, ConOps defines system use from a user—stakeholder perspective and provides a conceptual design outlining high-level architecture, key component specifications, and essential interactions required to achieve system objectives. A stakeholder is any individual, group, or organization that can affect or be affected by system’s development, deployment, operation, or maintenance (Freeman et al. 2018). Stakeholder participation may range from low-level information sharing to high-level collaboration (Brodrechtova et al. 2024; Fernandes et al. 2025).

The development of a ConOps generally follows three stages (Laarni et al. 2022; Sadrabadi et al. 2025). First, background and motivation are established by analyzing the current situation and its historical evolution. Second, an initial ConOps draft is produced by identifying user—stakeholder requirements, defining key operational scenarios, and outlining preliminary system architecture, including user–system interactions. Finally, the information is synthesized into a comprehensive ConOps document that defines operational tasks and scenarios, establishing a shared understanding of the envisioned system. While ConOps describes the intended system in detail, it does not prescribe methods for information elicitation, leaving their selection to the engineering or research team (Olayan et al. 2018).

## Materials and methods

This study proposes a methodology for developing CoOps document for a wildfire management platform within the SILVANUS project, which involved 49 partners from the EU, Australia, Indonesia, and Brazil. Over a 6-month period, methods for stakeholder engagement, data collection, and analysis were reviewed and selected to support the development of branch-specific ConOps addressing system goals, current situation, system requirements, and operational scenarios. The methodology was implemented across ten pilots in eight EU countries and Indonesia and selected based on project partners experience in wildfire management:

- Liburnija, Primorsko-Goranska Region, Croatia
- Moravian-Silesian Beskyds Mountains, Czech Republic
- La Jonchère-Saint-Maurice, Haute-Vienne, Nouvelle-Acquitaine, France
- Euboea (Evia) Island, Sterea Ellada, Greece
- Sebangau National Park, Central Kalimantan Province, Indonesia
- Gargano National Park, Italy
- Regional Natural Park of Tepilora, Italy
- Cova da Beira region, Portugal
- Rodna Mountains National Park, Romania
- Podpoľanie Region, Slovakia

### Information management: Guidelines preparation

**Fig. 1:**
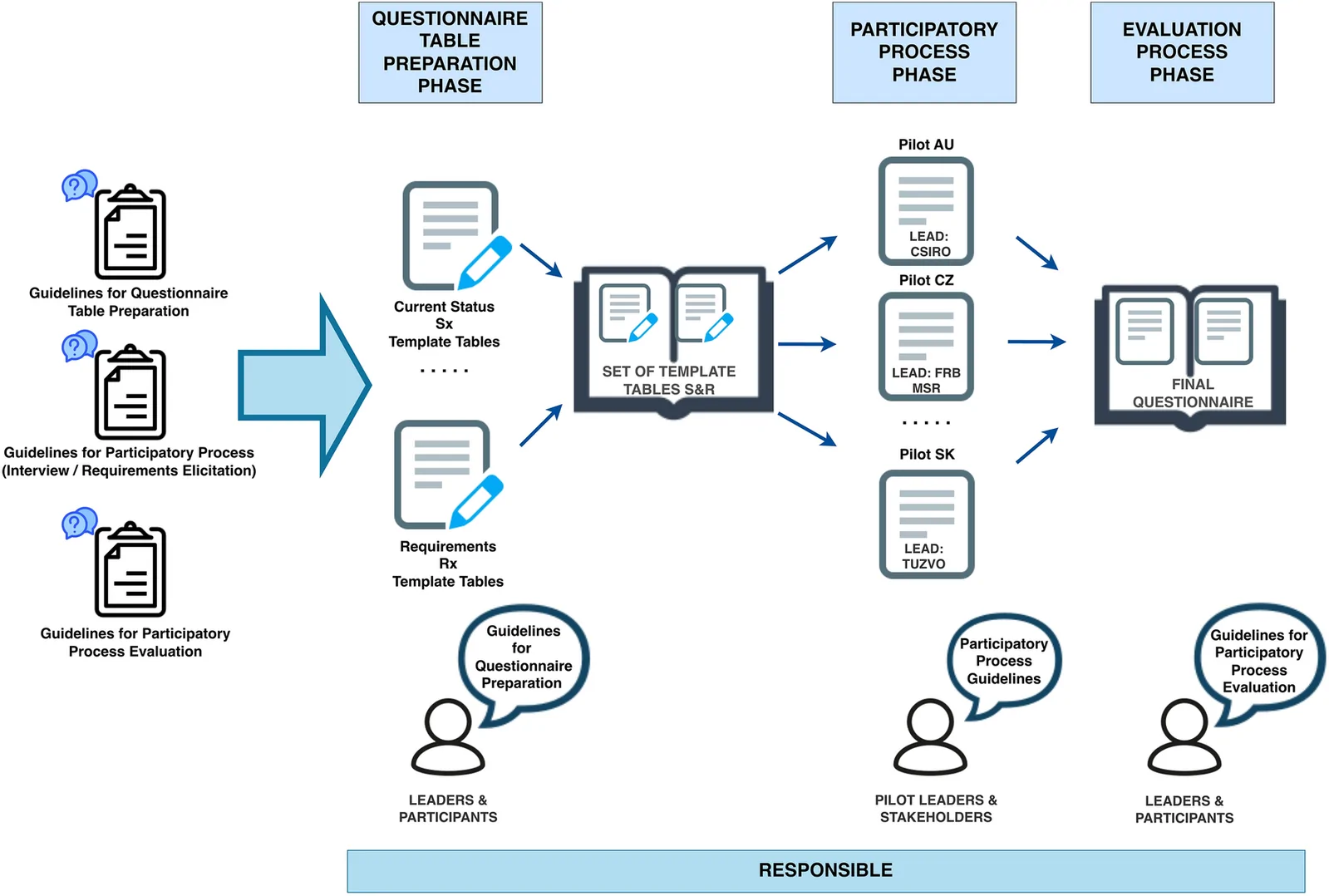
Guidelines preparation for information management to support ConOps development

Information management encompassing data collection, evaluation, and standardization, in the design of an integrated technological and information platform for wildfire management was supported through the development of common guidelines for questionnaires and participatory processes (Fig. 1). These guidelines assisted pilot leaders and stakeholders in formulating questions and collecting responses on existing datasets, current practices, and the functional requirements of the envisioned platform. Overall, the purpose was to ensure high-quality information management through structured guidance on questionnaire development, participatory process design, and evaluation.

**Fig. 2:**
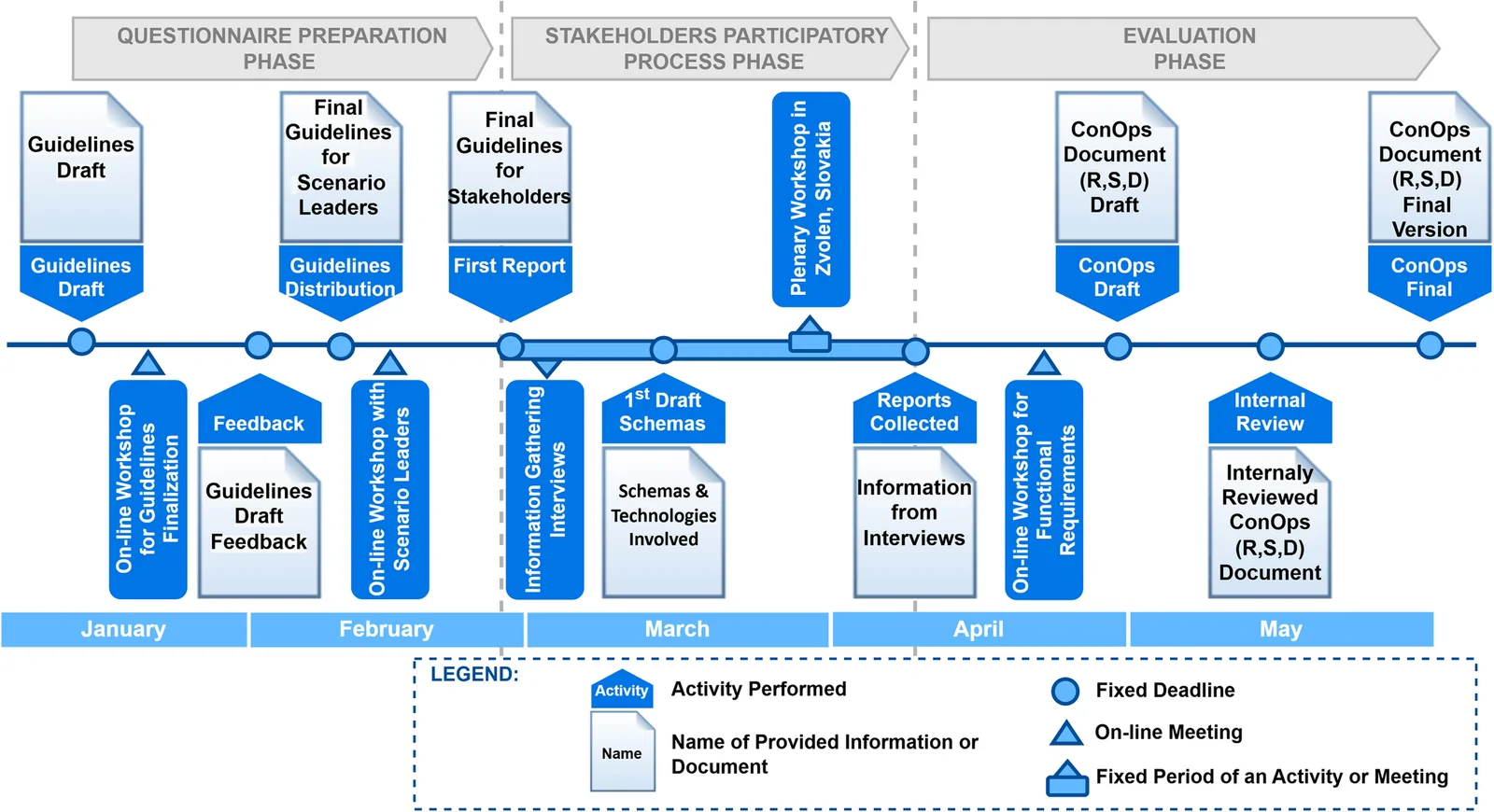
Phases of information management in ConOps development, enhanced through the participation of relevant stakeholders, including those from outside the project consortium

The development of the guidelines followed a structured, time-sequenced process (Fig. 2). Initially, pilot leaders and project partners were instructed on how to contribute to questionnaire creation and invited to review the draft to ensure alignment with the project’s professional objectives prior to finalization. Guidance was provided on how to engage relevant stakeholders to enhance participation in both the project and data collection. Stakeholder participation was defined, following Freeman (1984), as the active involvement of individuals or groups in decisions affecting them, particularly those engaged in wildfire prevention and response. Subsequently, the research team introduced the data collection methodology, such as interviews, to support consistent implementation across the project. To further assist the design and application of the guidelines, three online workshops were organized addressing key aspects of information management, including question design, implementation, consent procedures, operational scenario development (illustrated using the Slovakian pilot), and the use of the online survey tool JotForms for data submission (see “Information management: Stakeholder participation” section). Finally, the research team reviewed and incorporated feedback from pilot leaders and project partners to finalize the guidelines, ensuring a standardized and harmonized approach to questionnaire structure and data collection (Majlingová et al. 2022).

### Information management: Questionnaire preparation

**Fig. 3:**
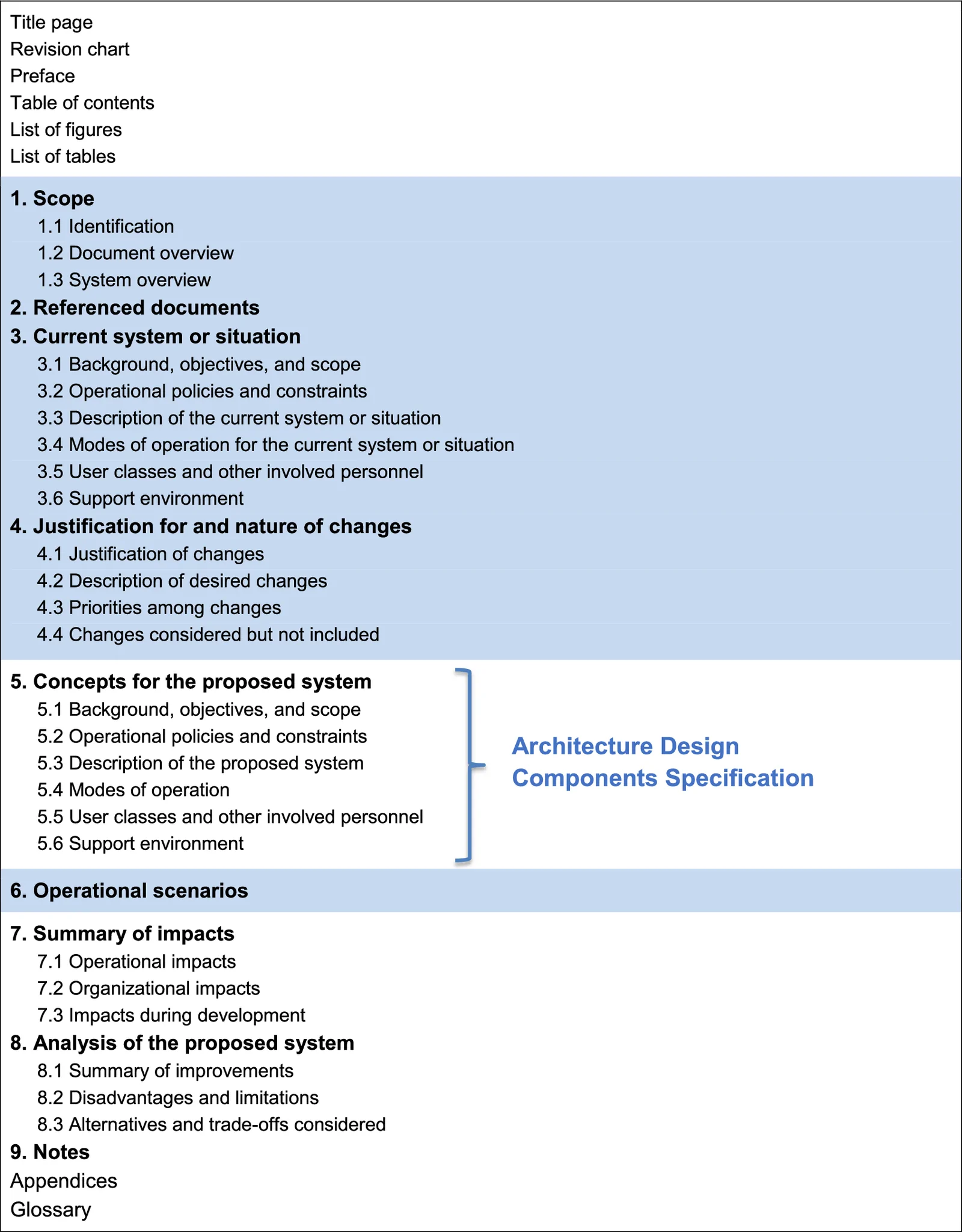
The generic structure of the ConOps document (*in *sensu IEEE 1998)

**Fig. 4:**
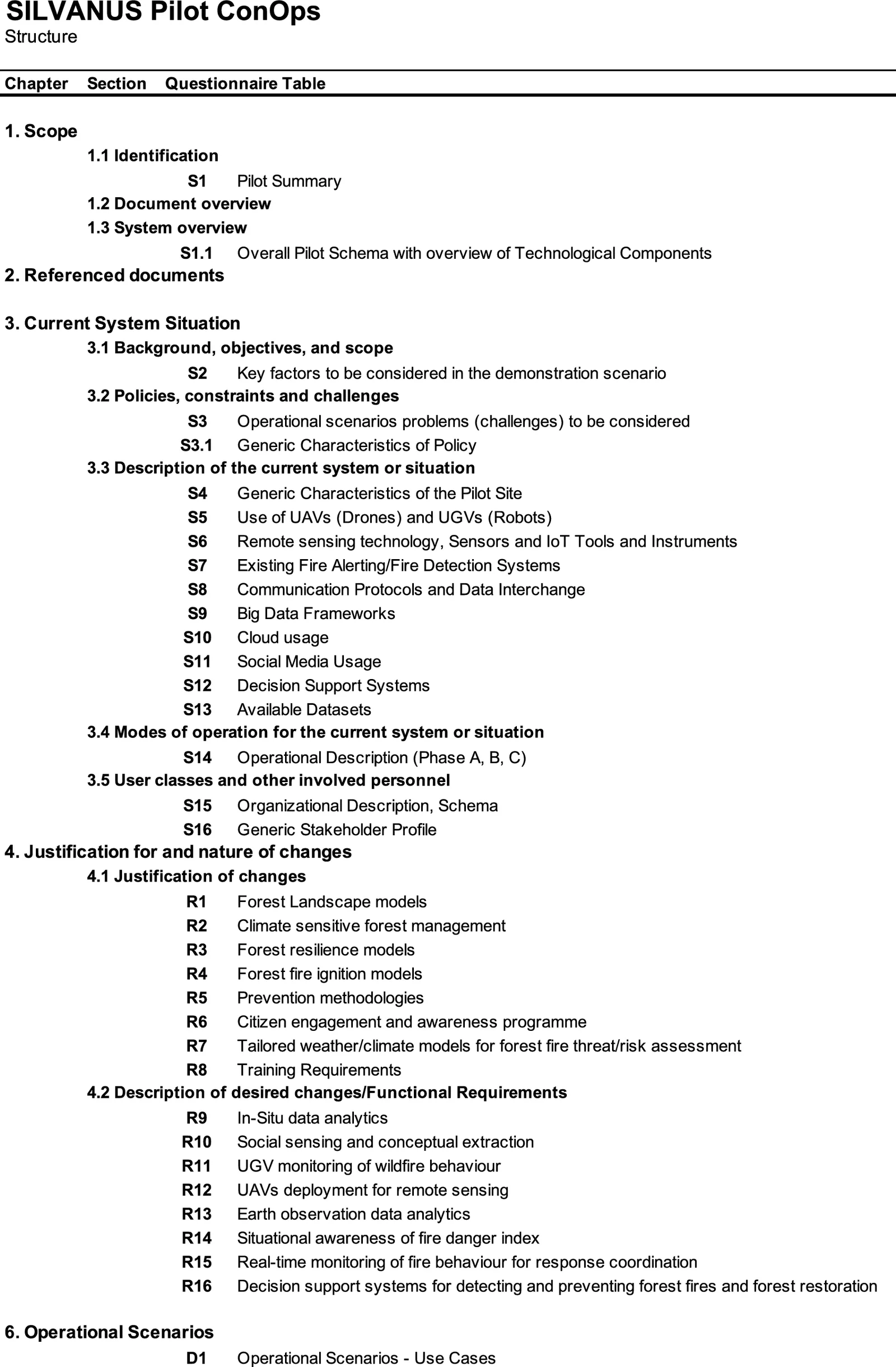
The questionnaire tables being sorted according to the generic structure of the ConOps document

The questionnaire comprised multiple thematic question sets addressing the existing status, functional requirements, and operational scenarios across all phases of wildfire management. Individual questions were structured in tables, and the questionnaire tables were organized into chapters that broadly followed the generic ConOps structure (Fig. 3). Tables S1–S16 contained open- and close-ended questions capturing pilot characteristics and current practices related to policies, forest landscape models, climate-sensitive forest management, forest resilience and fire ignition models, prevention methodologies, citizen engagement programs, weather and climate models for fire risk assessment, in situ data analytics, social sensing and concept extraction, UAV/UGV-based monitoring, earth observation data analytics, fire danger indices, real-time fire behavior monitoring for response coordination, decision support systems (DSS) for fire detection and prevention, and post-fire forest restoration (Fig. 4).

Tables R1–R18 summarized the stakeholders’ functional requirements concerning system’s objectives and functions, including wildfire and risk modeling, citizen engagement and awareness programs, social sensing and conceptual extraction, UAVs/UGVs deployment, and DSS. These requirements were collected using close-ended questions rated on a four-point Likert scale (“must,” “should,” “could,” and “won’t” be included in the system), supplemented by open-ended fields allowing respondents to provide qualitative justifications. All requirements were expressed in descriptive rather than technical terms, following Faulconbridge and Ryan (2002).

To capture contextual factors influencing these requirements, Tables D1–D2 were designed to collect information on internal (system-specific) and external (operational scenario) factors. Operational scenarios were defined as step-by-step descriptions illustrating how the system and its components operate and interact with users and external conditions. Precisely, scenarios identified fire incident locations across the ten pilots, the participating stakeholders, and both existing and newly proposed technologies (use cases) to be tested. Overall, they served to integrate system components, clarify user roles, and support activities such as simulation, testing, and user documentation (IEEE 1998).

To ensure manageability, the ConOps draft was divided into subdocuments intended for use at different stages of the system lifecycle and by various stakeholders. Structured according to the referenced ConOps schema, these subdocuments defined the current situation, stakeholder requirements, and operational scenarios for each pilot (see “Results” section).

### Information management: Stakeholder participation

Relevant stakeholders were purposively identified and selected by pilot leaders based on the characteristics of each wildfire management phase under investigation. Priority was given to stakeholders with direct professional involvement in wildfire management and situated within the same country, region, or organizational context. They represented a broad spectrum, including governmental agencies (e.g., fire and rescue services), civil society and community members (e.g., volunteer fire brigades, environmental non-governmental organizations, and municipalities), private sector organizations (e.g., forest and water management enterprises), and academic and research institutions, following the classification of Brodrechtova et al. (2024).

Initial engagement involved disseminating project information and objectives through leaflets and other promotional materials. Once contact was established, interviews were conducted with interested stakeholders either in person or via digital communication tools, using a standardized questionnaire (see “Information management: Questionnaire preparation” section). The questionnaire was initially developed in English and translated into the languages of the participating pilot countries. To ensure consistency in data reporting across pilots, responses were collected using an online version of the same questionnaire administered through the survey platform JotForm. The platform was selected for its ability to support structured input tables and formatted text fields, allow responders to resume partially completed surveys, and export responses in formats suitable for data analysis. Thus, although data collection involved in-person interactions, the standardized online questionnaire ensured harmonization across pilots, including uniform informed consent procedures, standardized data formats, and the availability of English translations.

### Data analysis and validation

A total of 61 scientists and experts from ten pilots participated in the online survey, and their responses were compiled into an MS Excel database. Although data were obtained from all pilot teams, several tables remained incomplete. According to informal feedback from pilot leaders, missing data resulted primarily from two factors: (i) limited capacity to complete the questionnaire, as the highly specialized questions required timely input from multiple stakeholders beyond the project consortium; and (ii) unavailability or restricted access to certain information, which was either unavailable during the survey period or accessible only for fee. To address these challenges, several measures were implemented during data collection. The research team provided online and in-person consultations to support questionnaire completion and stakeholder engagement, established multiple channels for data submission, and issued biweekly reminders to pilot leaders. Despite these efforts, the specialized nature of the required information, coupled with limited time and project partner capacity, resulted in some incomplete questionnaires.

The collected data were analyzed using structural content analysis based on deductive category application (Mayring 2022). The analysis employed a predefined set of categories derived from the questionnaire (see “Information management: Questionnaire preparation” section), which served as the analytical framework. Two complementary structuring techniques were applied. Content structuring, aimed at extracting and summarizing relevant information, was applied to assess current systems and operational scenarios. As data on current systems and operational scenarios were heterogeneous across pilots, and thus difficult to compare, they were summarized by identified technologies and across the three phases of wildfire management.

Scaling structuring, designed to define specific dimensions and characteristics using a four-point Likert scale, was used to assess functional requirements. To enhance the robustness of these findings, responses were validated by seven external stakeholders including two foresters, two fire engineering experts, and three civil protection specialists from Slovakia, the Czech Republic, and Poland. The same set of questions in form of R-tables was used during this validation process. All analytical procedures were conducted using MS Excel (Majlingová et al. 2022).

## Results

The first draft follows the generic ConOps structure, focusing on (a) the system and situation, (b) the justification for and nature of changes, and (c) operational scenarios.

### Wildfire management technology and services in use: Current system and situation

Across the ten pilot sites, fire detection and mapping primarily rely on satellite-based systems (e.g., Copernicus, EFFIS, and NOAA/NASA VIIRS) providing medium- to high-resolution spatial data (Table 1). The aerial platforms (manned aircraft) and UAVs complement satellite monitoring with higher spatial detail (down to ~ 15 cm) and flexible temporal coverage. However, spatial and temporal resolutions vary widely across pilots, and data access is often constrained by institutional or regulatory restrictions (e.g., Greece’s Copernicus activation authority). There is no unified data standard or integration mechanism across pilots for combining satellite, UAVs, and ground data in real-time. As a main problem is identified, lack of harmonized multi-source remote sensing data systems and consistent update frequencies. A variety of drone-mounted cameras and sensors (e.g., RGB, thermal, multispectral, and LiDAR) are used across pilots. Most pilots rely on DJI series drones (e.g., Matrice, Zenmuse, and Mavic) and in some cases by fixed camera systems (e.g., ForestWatch and Pelco Esprit). Data storage and management are mostly manual (e.g., SD cards), with limited real-time streaming or cloud-based integration. The main challenges include underdeveloped real-time image transmission, centralized data archiving, and the lack of a common data sharing platform. Some pilots use IoT sensor networks (e.g., Fire-Detect, weather stations, and FDAAS) to collect temperature, humidity, air pressure, wind speed, and soil moisture. These networks rely mainly on solar or battery power and connect via LoRa or mobile networks. However, sensor density and data accessibility vary; some systems are still in experimental phases. Overall, IoT networks remain fragmented and lack continuous coverage, system integration, and standardized data transmission protocols. Existing mobile applications (e.g., Provjeri, ROVKCU, and Mountain Rescue Service Slovakia) are primarily used for incident reporting, residents alerting, and sharing location data. These applications typically serve both the public and rescue services by providing weather forecasts, guidelines, and alerts. Most are free (no subscription) but remain limited in scope with respect to fire-specific data collection, coordination, and decision support. Generally, mobile applications are underutilized for citizen engagement and real-time situational awareness; and their integration with command-and-control centers is minimal. In few pilots, social media (e.g., Facebook) usage exists. Concerning wearable technology, only a few pilots named plans for health and safety monitoring of first responders, but no operational devices or systems are yet reported. Real-time monitoring of firefighter health, stress, and exposure conditions remains an unaddressed technological gap in majority of pilots.

**Table 1:** Summarized information on existing technology and services

Phase	Existing technology/service provided		Pilot
Pre-fire	Remote sensing technology	Satellite	Croatia, Greece, Indonesia, Italy, Portugal
		Aerial	Croatia, Portugal
		UAVs	Croatia, Czech Republic, France, Indonesia, Portugal, Slovakia
	Cameras		Croatia, Greece, Indonesia
	IoT devices		Greece, Portugal, Slovakia
	Mobile application		Slovakia
	Social media		France, Italy, Slovakia
Fire	Remote sensing technology	Satellite	Croatia, Greece, Portugal, Slovakia
		Aerial	Croatia, Portugal
		UAVs	Croatia, Czech Republic, France, Czech Republic
	Cameras		Croatia, Czech Republic, Greece
	IoT devices		Greece, Romania
	Mobile application		Romania, Slovakia
Post-fire	Remote sensing technology	Satellite	Greece
		Aerial	Slovakia

### Justification for and nature of changes: System requirements

The desired platform design should deliver key functionalities across multiple domains of wildfire management (Table 1). Core priorities include the provision of accurate information on fuel availability and fire danger indices. In the context of climate-sensitive forest management, the platform must support the visualization of climate statistics derived from historical data and future projections. To enhance forest resilience, it must prioritize the collection of data on fire ignition sources, including human activities, environmental conditions, and climate factors, enabling the development of probabilistic models to estimate wildfire ignition likelihood. Additionally, the platform must be capable of modeling fire behavior and simulating ignition scenarios to contribute to more effective prevention and preparedness strategies. The platform must include also social media or mobile applications that notify users of nearby fire events and enable them to report suspected fires using geolocation, images, and descriptive inputs. Results on training requirements indicate the need for a three-phase wildfire preparedness program, focusing on (i) training firefighters using VR/AR and UAVs/UGVs technologies, (ii) equipping incident commanders with fire behavior modeling and strategic decision support tools, and (iii) educating forest users on climate change impacts and sustainable land management. The platform must integrate micro-scale weather forecasting tailored to forest ecosystems and support data from a range of sources, including IoT devices, CCTV systems, and chemical sensors, enabling in situ environmental monitoring. UAVs/UGVs should be employed for remote sensing, mapping, and monitoring of wildfire dynamics. To support these capabilities, the platform must offer robust data filtering, integration, and processing functions. It must also provide DSS and scalable storage and repository infrastructure for managing heterogeneous data sources. Finally, the platform must deliver high-quality visualizations, including real-time monitoring of forest conditions and GIS/GPS-based mapping tools for operational coordination and fire response planning.

**Table 2:** Summarized information on functional requirements on the system

Phase	Functional requirements on desired system	Key technologies	Main outputs
Pre-fire	The wildfire management platform must integrate advanced tools for monitoring, prediction, and response. Core functionalities include the visualization of forest biodiversity, structural diversity, climate variables, and fuel conditions. It should assess fire danger, simulate ignition scenarios, and model human behavior (e.g., evacuation routes). The system should collect data from IoT sensors (e.g., air quality, humidity, temperature), UAVs/UGVs, and social media, applying AI/ML algorithms for fire detection and risk analysis. Citizen engagement may be supported through a mobile application for fire reporting and alerts. Additional requirements include integration, filtering, and storage of data for real-time forest landscape visualization, compliance with 5V data principles and a scalable architecture to support future development	AI/ML, UAVs/UGVs, IoT devices, social media, mobile application	Fire risk modeling, alerts, forest state visualization
Fire	The platform must enhance wildfire prediction, detection, response coordination, and health and safety monitoring. Key functionalities include climate and micro-weather visualization, fire ignition source analysis, and fire scenario simulation. The system must integrate data from IoT sensors, UAVs, and CCTV or cameras, applying AI/ML algorithms to detect fire risk, smoke, and high-risk areas. A mobile application could support real-time citizen alerts and fire reporting. DSS must guide fire spread prediction, response planning, and resource deployment. Additional capabilities should include health impact analysis related to wildfire emissions, integration with wearable devices, damage assessment, and GIS-based field visualization. Robust data processing and predictive modeling must ensure effective and coordinated wildfire management	DSS, AI/ML, IoT devices, CCTV, UAVs, GIS, mobile application	Fire detection, coordination tools, health monitoring
Post-fire	The platform must support long-term forest resilience and restoration by enabling visualization of landscape biodiversity, forest structural diversity, climate statistics, and fuel availability. It must provide forest resilience assessment models, including regeneration dynamics, and support continuous monitoring of forest restoration programs. It should allow stakeholders to explore alternative forest management and restoration through visualization tools. Effective integration and processing of heterogeneous data (e.g., weather and soil) from multiple sources are required to ensure the delivery of clean, reliable datasets	Data fusion, GIS/GPS, visualization tools, biodiversity assessment	Forest recovery mapping and visualization, DSS

### Operational scenarios

The operational scenarios are confined to ten SILVANUS pilot sites reflecting the direct participation of stakeholders and the existing and newly proposed technologies to be tested (Table 3). The Croatian pilot, located in Liburnija (Primorsko-goranska county), covers 30,800 ha, of which about 40% is forested. The area includes overgrown former pastures and agricultural land near residential zones, where wildfires occur mostly in spring threatening critical infrastructure and human safety. Stakeholders include government agencies (fire and rescue services, Ministry of Defense), civil society groups (volunteer fire brigades and firefighting associations), private sector actors (forest owners, forest management enterprises, and specialized technology providers), and university. The pilot aims to strengthen wildfire prevention and preparedness through public awareness, on-site surveillance, and microclimate data collection, while improving coordination among all actors through an integrated fire management system that combines monitoring, communication, and operational response. System module testing focuses, in phase A, on fire detection systems, camera-based monitoring, and across phases A/B on operational response and technology deployment such as UAV-based mapping and reconnaissance, inter-UAV communication, and fire spread forecasting.

**Table 3:** Summarized information on use cases planned in operational scenarios

Phase	Use cases		Country
Pre-fire	Mapping, risk, and environmental assessment	Mapping of pilot areas	Croatia, France, Greece, Italy, Slovakia
		Fire hazard, danger, susceptibility, vulnerability and risk mapping	Croatia, Greece, Italy, Portugal, Romania, Slovakia
		Territory opening-up analysis (GIS for fire truck routes)	Slovakia, Romania
		(Self-) assessment toolkit for environmental modeling, biodiversity	Romania
	Technological prevention and monitoring	Use of UAVs/UGVs, surveillance cameras, IoT sensors for monitoring	Croatia, France, Greece, Italy, Portugal, Romania, Slovakia
		Analysis of area for sensor deployment (temperature, humidity, counting)	Romania
		Development of high-resolution land cover maps, vegetation and livestock grazing monitoring near critical infrastructure	Portugal
	Preparedness and capacity building	Public awareness and education campaign	Croatia, Czech Republic, France, Greece, Italy, Portugal, Romania, Slovakia
		Guidelines for training firefighters, (GIS supported) fire prevention plan	Croatia, Greece, France, Italy, Slovakia
		Non-technological preparedness (e.g., infrastructure, evacuation planning)	Greece
		Review of national/EU regulations on fire management	Portugal
Fire	Early detection and situation awareness	Early warning via satellites, drones, CCTV, social media, mobile applications	Czech Republic, Croatia, Greece, Slovakia
		Fire sighting patrols, reporting via social media and mobile application	Italy, Slovakia
		Social sensing algorithms for fire detection and evolution	Greece
		Real-time data transfer to operation command centers	Romania
	Monitoring, modeling and DSS	Using UAVs (swarm drones)/UGVs for mapping temperature, composition of combustion gases, fire spread, human behavior	Czech Republic, France, Italy, Romania, Slovakia
		Mapping of suitable water sources	Greece, Romania, Slovakia
		Fire propagation modeling	Croatia, Greece, Italy, Slovakia
		Using IoT sensors for real-time modeling (e.g., microclimate data)	Croatia, Slovakia
		DSS for emergency management	Czech Republic, Croatia, Greece
	Command, coordination, and communication	Mobile command centers	Greece, Slovakia
		Incident management with GINA mobile application	Czech Republic, Croatia, Slovakia
		Navigation to fire sites and firefighter tracking	Czech Republic, Slovakia
		Mesh-in-the-sky system for communication	Croatia, Slovakia
		Deployment of resources of various organizations according to emergency plans	Greece, Slovakia
	Active firefighting operations	Deployment of UGVs for transport and firefighting purposes	Czech Republic, Croatia, Slovakia
		Deployment of aerial forces (helicopters)	Croatia, Slovakia
		Implementation of protection and evacuation measures	France, Italy
		Monitoring firefighters’ health and safety	Greece, Slovakia
Post-fire	Forest modeling, planning, monitoring, and restoration	Sustainable forest management and silvicultural optimization	Greece, Italy, Portugal, Slovakia
		Forest growth models and management optimization	Greece, Slovakia
		Building historical databases on fires and restoration outcomes	Indonesia
		Soil protection and restoration (short and long term)	Greece, Indonesia
		Mapping/surveillance of land use changes, biodiversity, and burnt areas, (IoT sensors, CCTV)	Greece, Indonesia, Italy
	Citizen engagement	Citizen engagement via mobile application	Greece, Indonesia

The Czech Republic pilot is based in the Moravian-Silesian Beskyds Mountains, covering 116,000 ha. The area is frequently affected by wildfires and other natural disasters, including strong winds, floods, and landslides. Key stakeholders include government agencies (fire and rescue and emergency services), civil society organizations (volunteer fire brigades and municipalities), businesses and industry (forest owners, forest management enterprises, specialized technology providers), and university. The expected outcomes of the pilot include, in phase A, enhanced public awareness and citizen training, and in phase B, improved wireless communication infrastructure, deployment of UAVs/UGVs for fire surveillance, and the use of DSSs for resource allocation and emergency response. Accordingly, system testing focuses on phases A/B and contains fire spread modeling, citizen engagement tools, and the deployment of UAVs/UGVs for early detection, firefighting support, rescue operations, and logistic coordination.

The French pilot is in La Jonchère-Saint-Maurice (Nouvelle-Acquitaine), a wildfire-prone area near sensitive sites where dense smoke and strong winds (> 70 km/h) complicate firefighting operations. Stakeholders include government agencies, notably firefighting services, and specialized technology providers in the project. In phase A, pilot objectives encompass enhanced monitoring tools, increased public awareness, and the use of a fire reporting application, in phase B, sensors and satellite data testing, reduced response time, and real-time digital control of interventions, and in phase C, multi-stakeholder engagement in post-fire reforestation and monitoring. The system is deployed through two modules. Phase A comprises VR/AR-based firefighter training, a citizen-focused mobile application, and initial fire detection systems. Phase B covers operational response and technology deployment like UAV/UGV-based mapping and reconnaissance, fire spread forecasting for command support, real-time coordination interfaces, fire suppression and safety equipment, and firefighter operations and technology demonstrations.

Greece pilot is based in Evia (Sterea Ellada), a region experiencing frequent and intense wildfires, particularly during summer months. The 2021 mega-fire, which burned over 500,000 ha, highlighted the areas’ extreme vulnerability. While most fires are small, the combination of dry summers, strong winds, and flammable lowland pine forests increase wildfire risk. Key stakeholders include government agencies (fire and rescue services, first responders, civil protection, and forest authorities), civil society groups (volunteers and local citizens and specialized technology providers), and universities. Pilot objectives are phased as follows: phase A focuses on fire prediction models, public awareness and education, and citizen engagement tools, phase B on enhanced response coordination, and phase C on restoration planning and the assessment of ecosystem quality and habitat recovery. System testing includes, in phase A, fire spread forecasting integrated with evacuation and health-planning tools, citizen engagement and biodiversity profiling applications, across phases A/B, deployment of fire detection technologies (e.g., IoT devices and social sensing) and real-time response coordination systems, and in phase C, citizen-led restoration initiatives and soil rehabilitation monitoring.

The Indonesian pilot is implemented in Sebangau National Park, covering 568,700 ha of peat swamp forest. Wildfires pose the primary threat to the area, causing severe ecological impacts, particularly during the dry season and El Niño years, which typically occur on a 5-year cycle. Key stakeholders include governmental agencies (national park authorities), civil society members, specialized technology companies participating in the project, and academic institutions such as universities. The pilot focuses primarily on post-fire soil rehabilitation. Accordingly, system module testing includes soil rehabilitation strategies, citizen engagement applications, biodiversity profiling tools, and fire spread forecasting.

The Italian pilot takes place in the Parco Naturale Regionale di Tepilora, an 8,000 ha protected area in Sardinia facing high wildfire and hydrogeological risks in summer, with about 60% of wildfires caused by arson and remainder by accidents such as discarded cigarette butts and stubble burning. Key stakeholders include government agencies (forests agencies and national park guards), civil society organizations and community members (municipalities, volunteer groups, and environmental NGOs), and specialized technology providers participating in the project. The pilot pursues three phased objectives: in phase A, wildfire prevention through early detection in high-risk areas, deployment of monitoring tools, and public awareness raising, across phases A/B, improved response planning, communication, and stakeholder coordination, and in phase C, habitat restoration and conservation in line with legal requirements. Accordingly, system testing includes, in phase A, development of fire management plan, public awareness activities, citizen engagement applications, and satellite-based fire prevention technologies, across phases A/B, the testing of operational response technologies, including UAV-based mapping and reconnaissance, IoT devices, social sensing, and fire spread forecasting, and in phase C, the development of resilience strategies and restoration guidelines.

The second Italian pilot is implemented in the Parco del Gargano, covering 120,000 ha. The region experiences frequent wildfires during the summer, predominantly caused by intentional ignitions (70%) and negligence (30%), with no recorded natural causes. Fire risk is concentrated along coastal slopes characterized by diverse vegetation with pine forests. The pilot involves various stakeholders like governmental agencies (civil protection services, forestry administration, and national park authorities), civil society organizations (volunteer groups), specialized enterprises participating in the project, and educational institutions (local schools). In phase A, objectives include improved monitoring tools and increased public awareness. Across phases A/B, the focus is on the deployment of sensors and advanced fire prevention technologies, such as satellite monitoring. Phase C emphasizes stakeholder engagement in ecological restoration and rehabilitation. Thus, system module testing contains, in phase A, fire risk assessment and fire detection using IoT devices and citizen engagement mobile applications, across phases A/B, operational response and technology deployment involving IoT devices, social sensing, and fire spread forecasting, and in phase C, ecological restoration through reforestation with native species and UAV-based surveillance.

The Portugal pilot is in Cova da Beira within a 500-ha agroforestry farm, having 200 ha Pyrenean oak forest. The area faces wildfire risk due to the combined effects of climatic conditions, rugged terrain, and pine and eucalyptus monocultures, further compounded by human practices such as pasture burning. Stakeholders comprise government agencies (fire and rescue services and forestry administration), civil society organizations (agroforest associations and volunteer groups), private sector (emergency and construction firms, water infrastructure operators, and specialized enterprises involved in the project), and universities. Pilot outcomes are phased as follows: phase A focuses on fire hazard monitoring, citizen engagement through awareness programs and mobile applications, and planning cost-efficient interventions, phase C on balancing grazing management with biodiversity and soil protection while evaluating restoration impacts. System module testing in phase A includes biodiversity profiling application, citizen engagement programs/applications, and fire detection technologies (e.g., fire risk assessment tools, UAVs/UGVs for data collection and mapping, and IoT devices).

The Romanian pilot is implemented in Rodna Mountains National Park, covering 47,177 ha. Wildfire occurs mostly in spring and summer, mainly due to agricultural burning and human negligence, with risk further amplified by seasonal increases in tourism along hiking trails and camping areas. Stakeholders include government agencies (fire and rescue services and national park authorities), civil society groups (forest associations), and specialized enterprises providing technological solutions. In phase A, the pilot focuses on wildfire risk identification, implementation of prevention measures, improvement of public communication, and delivery of AR/VR-based training to enhance preparedness. Across phases A/B, objectives include strengthening institutional coordination, optimizing resource use, and shifting toward technology-supported response mechanisms, such as UAV- and satellite-based early fire detection. Therefore, system testing in phases A/B includes AR/VR training modules, public awareness tools, fire spread forecasting, and fire detection technologies (e.g., IoT devices). In phase B, testing further extends to real-time mapping of emergency access routes and water sources, with data transmitted to the Fire and Rescue Command Centre.

The Slovakian pilot is in the Podpoľanie region, covering 67,000 ha, of which forests account for about 45%, alongside grasslands and agricultural land. The area experiences recurrent wildfires, often caused by intentional burning of dry grass and crop residues in spring and autumn, which can spread into nearby forests. Stakeholders include government agencies (fire and rescue services, armed forces, fire brigade, forest, and environmental authorities), civil society (volunteer fire brigades, environmental NGOs, and municipalities), businesses and industry (forest owners, forest management firms, media, and specialized technology providers), and university. Pilot aims are phased as follows: in phase A on fire risk assessment, evaluation area accessibility, and enhanced monitoring, in phase B on deployment of real-time surveillance technologies (e.g., UAVs), and in phase C on application of sustainable forest management fostering multi-stakeholder engagement and consensus. System module testing includes, in phase A, risk assessment, monitoring using CCTV, UAVs, IoT devices, and GIS-supported operational planning (e.g., mapping of water sources, heliports, and fire trucks access routes), across phases A/B, early fire detection through CCTV, crowdsourcing applications, and UAV/UGV/helicopter deployment for detection and firefighting, fire spread forecasting and health status mapping, and in phase C, forest growth simulations for long-term planning.

## Discussion

The proposed methodology for preparing ConOps and its application across ten international pilots represents a first step in designing the wildfire management platform developed under the EU project SILVANUS. The approach integrated standardized information management, participatory engagement, and cross-sectoral collaboration that are critical for designing complex, data-driven environmental management platforms. The iterative, multi-phase ConOps development process revealed both the strengths and challenges of applying systems engineering methods within a real-world, multi-country context. While structured information management and stakeholder involvement in operational scenario design and requirements elicitation enabled the systematic incorporation of diverse perspectives, the iterative refinement cycles were resource-intensive and time-consuming, reflecting the platform’s broad scope and the inherently text-heavy nature of ConOps documentation.

Data collection on current systems and contextual conditions emerged as a major challenge. Despite the establishment of harmonized guidelines and standardized questionnaires, available information remained fragmented or, in some cases, entirely unavailable. Improving response rates would have required additional time, resources, and deeper engagement from pilot-level partners. Similar challenges have been noted by Mostashari et al. (2012), who identified data fragmentation in one-third of the ConOps-related studies they reviewed. Two key lessons emerged. First, baseline data on existing systems and capabilities must be prioritized early in the systems engineering lifecycle; when deferred, data collection becomes disproportionately time- and resource-intensive. Second, because ConOps documentation defines the system from a user–stakeholder perspective, participatory processes must be embedded at the earliest stages of project design. Delayed or insufficient engagement risks inconsistencies between stakeholder expectations and technical implementation, potentially introducing bias into platform development. These findings reaffirm the argument of Fairley and Thayer (1997) that ConOps documentation evolves dynamically alongside the system, adapting to emerging information and stakeholder needs.

Applying the proposed methodology across ten pilot sites resulted in the first draft of ConOps documentation, representing a major step toward an integrated wildfire management platform. Comparison of the current situation, functional requirements, and operational scenarios demonstrates alignment between existing system deficiencies and proposed technological and operational advancements. Existing wildfire management systems are characterized by fragmented technologies, inconsistent data standards, and limited interoperability across satellites, UAVs, IoT devices, and ground-based monitoring platforms. Real-time data integration remains limited, access to high-resolution satellite products is restricted, and citizen engagement tools are underutilized. Health and safety monitoring for first responders is largely absent, and centralized data management is underdeveloped. In response, the identified functional requirements emphasize creation of integrated, interoperable platform capable of fusing multi-source data, supporting AI-driven fire detection and behavior modeling, and providing real-time visualization and decision support. Core functionalities include predictive fire modeling, microclimate forecasting, post-fire recovery assessment, and citizen participation through mobile applications, while wearable sensors and cloud-based systems are proposed to enhance responder safety and operational coordination (e.g., Balogh et al. 2023; Chandramouli et al. 2025).

Phase-specific demands are embedded within the proposed framework. In the pre-fire phase, stakeholders prioritized early fire prediction and prevention through the integration of UAVs/UGVs, IoT networks, and citizen reporting mechanisms, consistent with recent studies emphasizing social participation in early warning systems (e.g. Gatial et al. 2024; Valente et al. 2024; Arvandi et al. 2025). During the active fire phase, demands focused on real-time communication, data-driven decision-making via DSSs, and coordinated response based on live data from UAVs, ground sensors, and weather models, reflecting advances in UAV swarm deployment and AI-based analytics in wildfire management (e.g. Zelenka et al. 2023; Boroujeni et al. 2024; Sadrabadi et al. 2025). In the post-fire phase, attention shifted toward ecosystem recovery and long-term resilience, requiring tools for forest restoration mapping, resilience modeling, and DSS based on validated and integrated datasets, in line with contemporary restoration strategies (e.g., Nelson et al. 2024; O’Mara et al. 2024; Zhai et al. 2025).

Operational scenarios across pilots in Europe and Indonesia illustrated the practical implementation of these concepts. UAVs, IoT networks, and DSSs have been deployed for early fire detection, resource allocation, and coordinated response. VR/AR training, fire spread modeling, and biodiversity monitoring further demonstrate the platform’s capacity to support prevention, mitigation, and restoration. However, institutional coordination and multi-stakeholder governance remain cross-cutting challenges (Eckerberg and Buizer 2017; Kete 2023; Brodrechtova et al. 2024). Existing systems continue to operate within fragmented institutional structures, with limited collaboration among governmental agencies, emergency services, and research organizations. Although the functional requirements envisioned a unified platform enabling interoperability and shared decision-making, governance aspects were not yet fully embedded within the pilot operational scenarios.

In summary, the first draft of the ConOps documentation provides a coherent blueprint for an integrated wildfire management platform that addresses technological, operational, and social dimensions, while also revealing persistent challenges in governance and multi-stakeholder coordination. Although established standards for ConOps documentation exist, tailoring them to specific user needs remains a significant challenge. The proposed methodology supported the development of a robust, stakeholder-driven system aligned with operational realities and responsive to the challenges posed by wildfires. A key contribution of this approach lies in the systematic identification of essential system modules and operational scenarios. However, validating its effectiveness relative to traditional approaches requires testing the system and its components in real-world operational contexts (Chandramouli et al. 2025). This underscored the need for context-sensitive technology design and capacity building measures, particularly given that, while developed countries have made notable progress in this area (e.g., Kalapodis et al. 2025), the adoption of technological innovations in developing countries remains constrained by socio-economic and institutional barriers (e.g., Kilawe et al. 2021; Cassidy et al. 2022; Nunoo et al. 2024). Conducting such tests is therefore essential, as unforeseen interactions among system components may affect both the accuracy of the ConOps documentation and the overall performance of the wildfire management platform.

## Conclusion

The development of the ConOps within the SILVANUS project demonstrated the effectiveness of a structured and participatory approach to designing an integrated wildfire management platform. As emphasized by O’Mara et al. (2024) and Zhai et al. (2025), integrating all three wildfire management phases within a single, cohesive system is essential to support complex decision-making processes spanning prevention, response, and recovery. Engagement of stakeholders across ten pilot sites enabled the identification of operational needs and functional requirements across all wildfire phases, linking current system deficiencies with proposed advances in data integration, AI-driven analytics, and decision support. However, while ConOps documentation serves as a strategic tool for platform development, its broad scope introduces challenges related to limited technical guidance, insufficient stakeholder cooperation, and the resource-intensive nature of iterative refinement. These findings stress the need for early baseline data collection and sustained stakeholder engagement throughout the design process. Addressing gaps in governance, data management, and stakeholder coordination will be crucial to achieve a fully integrated, resilient, and adaptive wildfire management platform. Overall, the ConOps methodology proved instrumental in aligning technological innovation with user-centered operational realities and offers a replicable framework for future large-scale environmental management initiatives.

## Supplementary Information

Below is the link to the electronic supplementary material.

Supplementary file1 (PDF 289 KB)

## Data Availability

Available from the corresponding author upon a reasonable request that does not contradict the consent for the survey.

## Footnotes

1 https://silvanus-project.eu.
