# Supplementary material for: Formulating a concept of operation (ConOps): A stakeholders-assisted standardization process for developing an integrated wildfire management platform
Source: Ambio. 2026 May 3;55(10):2516–32. doi: 10.1007/s13280-026-02375-1 (PMC13554012; doi:10.1007/s13280-026-02375-1)
Supplement: Supplementary file 1 [file 13280_2026_2375_MOESM1_ESM.pdf]

Electronic supplementary material

Title:
Formulating a Concept of Operation (ConOps): A stakeholders-assisted standardization process for developing an integrated wildfire management platform

Journal:
Ambio

Overview of supplementary material:

Table S1. Products and technologies with verified references and URLs

Figure S1. Visualization of the analysis of justifications for and the nature of changes across the pre-fire (A), during-fire (B), and post-fire (C) phases, organized according to the R1-R16 questionnaire tables, excluding R8, which contained an open-ended question. Cross-validation (CV) outcomes are included to assess the robustness of the results (*in sensu* Majlingová et al. 2022).

Table S1. Products and technologies with verified references and URLs

Product / Technology Name	Reference (Academic style + URL)
DJI Matrice 4 Series	DJI (2025). Matrice 4 Series – Enterprise drone platform. DJI Innovations. Retrieved from https://enterprise.dji.com/matrice-4-series
DJI Zenmuse Series	DJI (2025). Zenmuse camera & gimbal series. DJI Innovations. Retrieved from https://www.dji.com/products/compare-zenmuse
DJI Mavic 4 Pro	DJI (2025). Mavic 4 Pro – Consumer drone platform. DJI Innovations. Retrieved from https://www.dji.com/global/mavic-4-pro
ForestWatch	EnviroVision Solutions (2025). ForestWatch® early wildfire detection system. Retrieved from https://evsolutions.biz/forestwatch/
Pelco Esprit (Enhanced)	Pelco (2025). Esprit Enhanced pressurized PTZ camera. Retrieved from https://www.pelco.com/cameras/ptz/esprit-enhanced-pressurized
Copernicus Programme	European Commission (2025). About Copernicus. Retrieved from https://www.copernicus.eu/en/about-copernicus
EFFIS (European Forest Fire Information System)	European Commission JRC (2025). EFFIS – European Forest Fire Information System. Retrieved from https://forest-fire.emergency.copernicus.eu/
NASA VIIRS Active Fire Data	NASA Earthdata (2024). VIIRS I-Band 375 m Active Fire Data. Retrieved from https://www.earthdata.nasa.gov/data/instruments/viirs/viirs-i-band-375-m-active-fire-data
Semtech LoRa	Semtech (2025). LoRa Technology Overview. Retrieved from https://www.semtech.com/lora
Campbell Scientific Automatic Weather Stations	Campbell Scientific (2025). Automated Weather Stations – Technical overview. Retrieved from https://www.campbellsci.com/automated-weather-stations
FLIR K-Series Firefighting Cameras	Teledyne FLIR (2025). K-Series Firefighting Thermal Imaging Cameras. Retrieved from https://www.flir.com/en-eu/browse/first-responders/firefighting-cameras/kxx-series/
MicaSense Multispectral Cameras	MicaSense (2025). Comparison of MicaSense Cameras. Retrieved from https://support.micasense.com/hc/en-us/articles/1500007828482-Comparison-of-MicaSense-Cameras
RIEGL Airborne LiDAR	RIEGL (2025). Airborne Laser Scanners (ALS). Retrieved from https://www.riegl.co.uk/airborne-laser-scanners
Varjo VR/XR Training	Varjo (2025). Training and Simulation – Use Cases. Retrieved from https://varjo.com/use-cases/training-and-simulation
Esri ArcGIS Platform	Esri (2025). ArcGIS – Geospatial platform overview. Retrieved from https://www.esri.com/en-us/arcgis/geospatial-platform/overview
IQ FireWatch	IQ FireWatch (2025). Automated early wildfire detection system. Retrieved from https://www.iq-firewatch.com/
OroraTech Wildfire Solution	OroraTech (2025). Wildfire Solution – Satellite-based wildfire intelligence. Retrieved from https://ororatech.com/all-products/wildfire-solution
Dryad Silvanet Wildfire Sensor	Dryad Networks (2025). Silvanet Wildfire Sensor. Retrieved from https://www.dryad.net/wildfiresensor
Amplicam Wildfire Guardian	Amplicam (2025). AI Wildfire Detection & Early Warning System. Retrieved from https://amplicam.ai/solution/
RoboticsCats AI Wildfire Detection	RoboticsCats (2025). AI Wildfire Detection System. Retrieved from https://roboticscats.com/
Bosch AVIOTEC 8000i IR	Bosch Security (2025). AVIOTEC – Fire & smoke detection camera. Retrieved from https://www.boschsecurity.com/en/products/fire-and-smoke-detection-camera/
SenseNet AI Wildfire Detection	SenseNet (2025). AI Mountain and Forest Fire Detection. Retrieved from https://www.sensenet.ca/
Forest Radar – Fire Monitoring App	Forest Radar (2025). Fire Monitoring Application. Retrieved from https://www.forestradar.com/products/fire-monitoring-app
Jotform (online forms)	Jotform (2025). Free Online Form Builder & Creator. Retrieved from https://www.jotform.com/

Figure S1. Visualization of the analysis of justifications for and the nature of changes across the pre-fire (A), during-fire (B), and post-fire (C) phases, organized according to the R1-R16 questionnaire tables, excluding R8, which contained an open-ended question. Cross-validation (CV) outcomes are included to assess the robustness of the results.

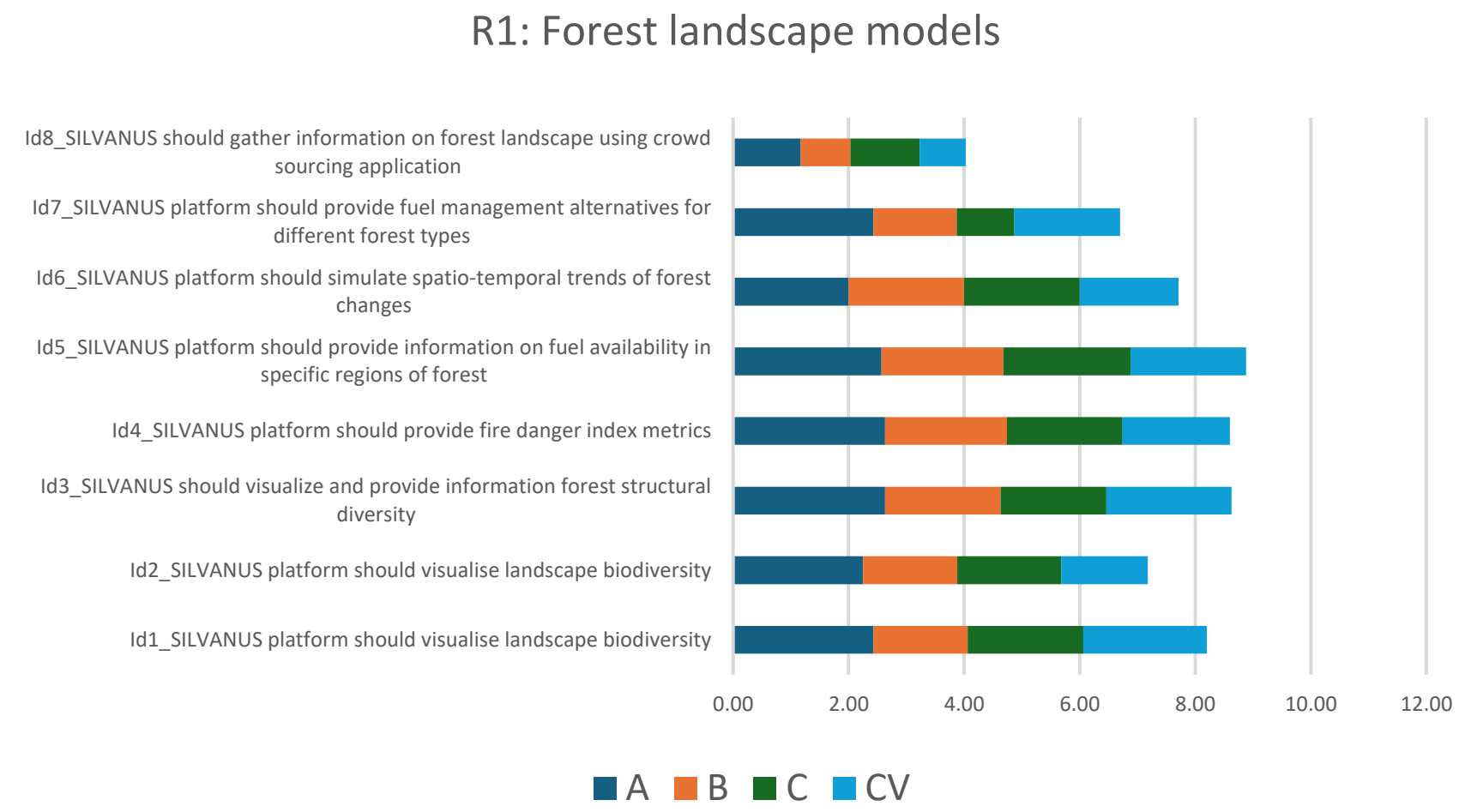

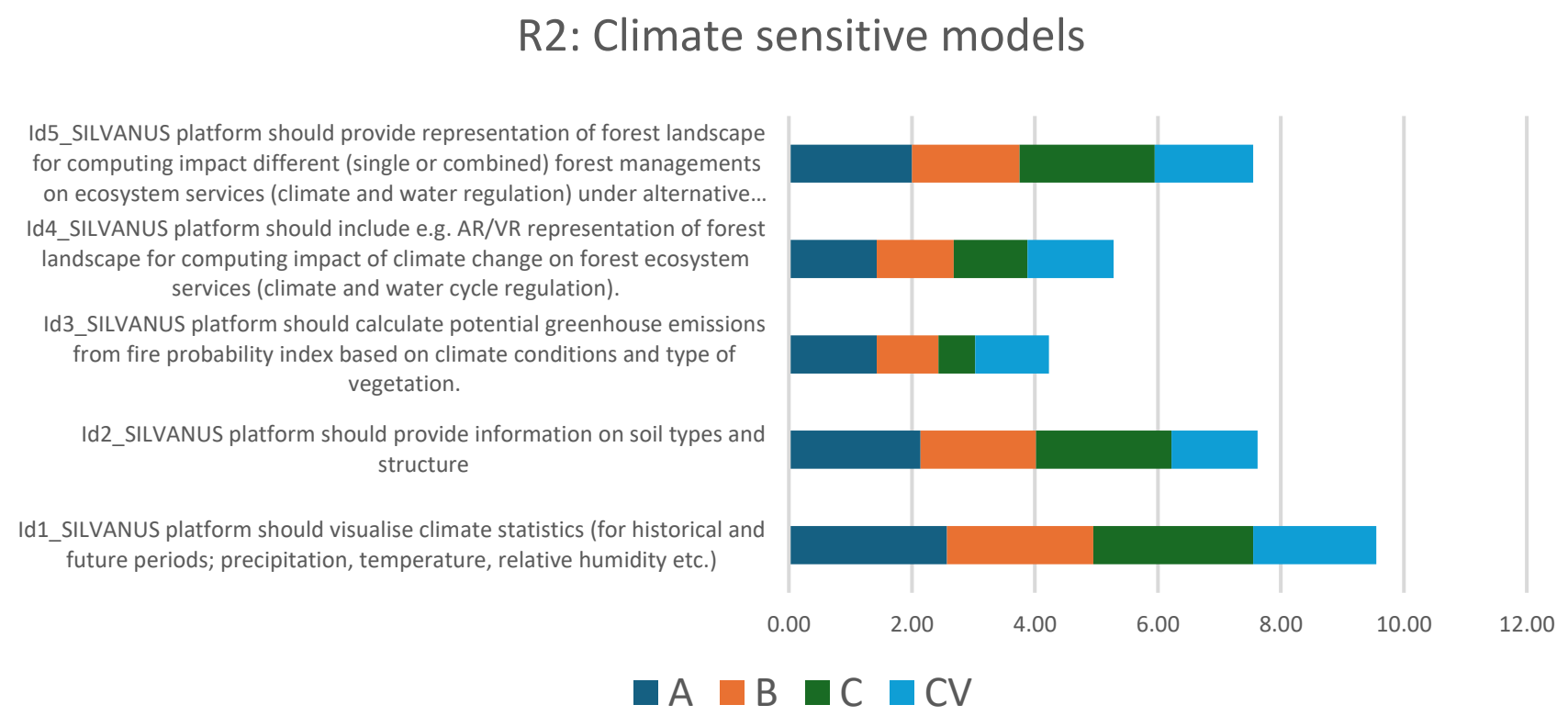

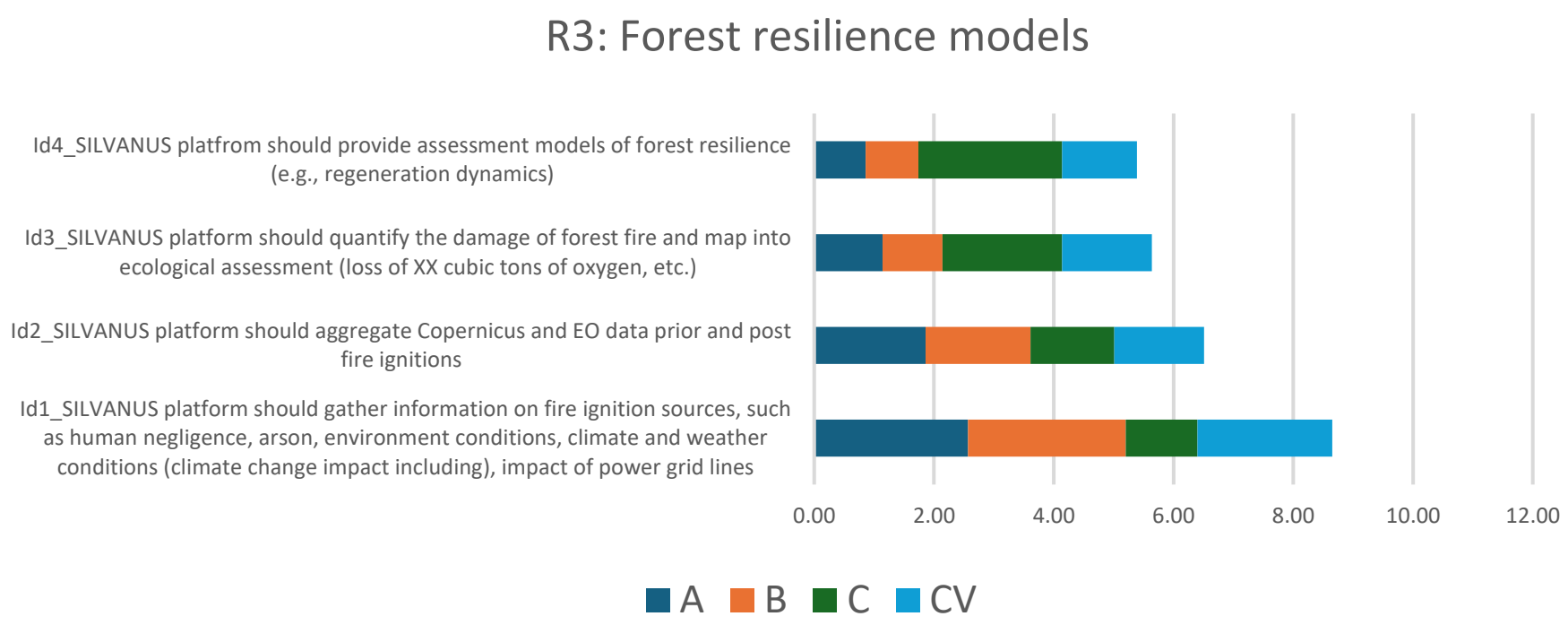

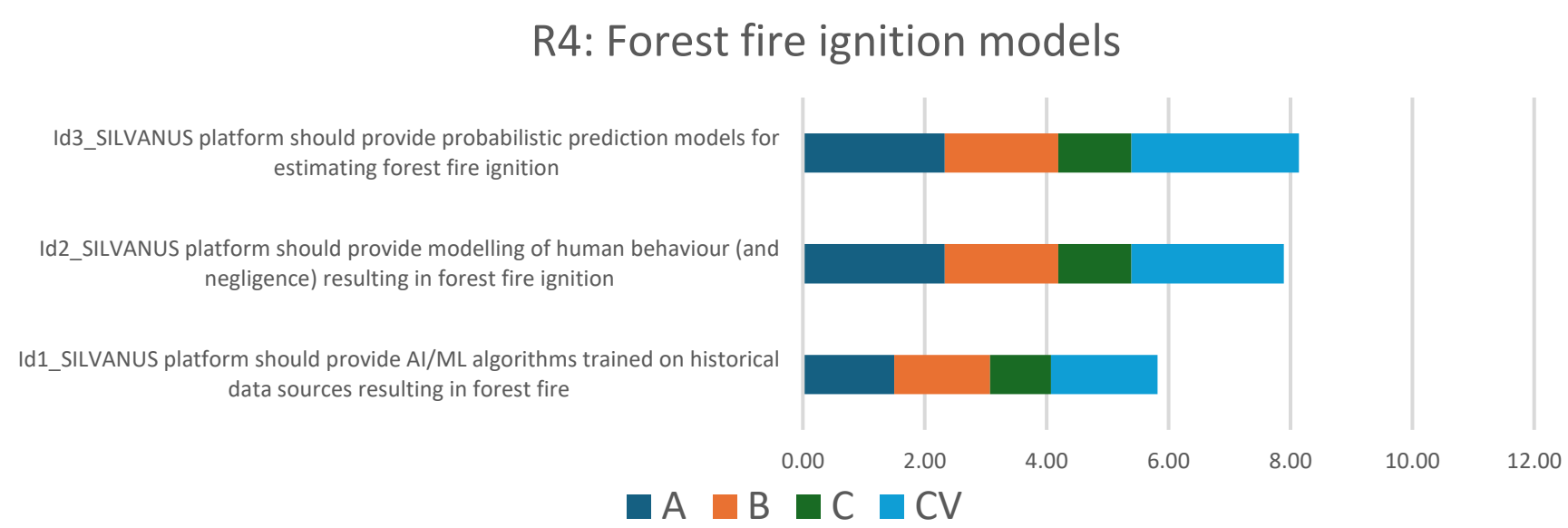

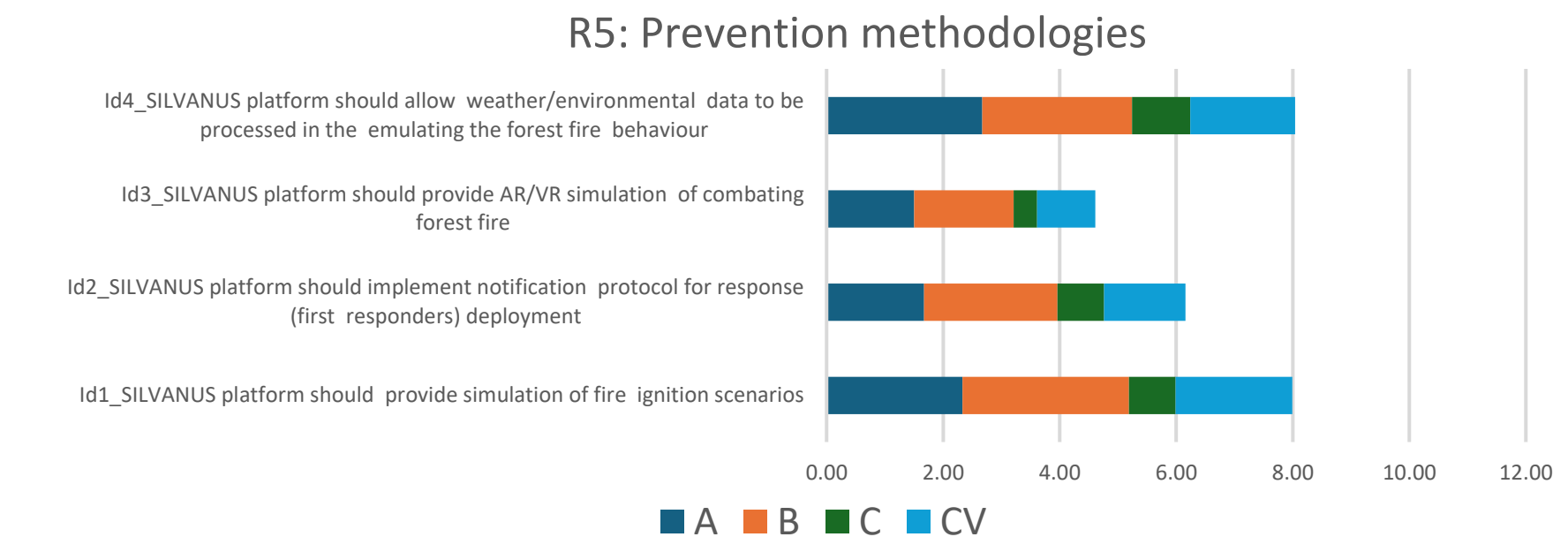
R5: Prevention methodologies
Id4_SILVANUS platform should allow weather/environmental data to be processed in the emulating the forest fire behaviour
Id3_SILVANUS platform should provide AR/VR simulation of combating forest fire
Id2_SILVANUS platform should implement notification protocol for response (first responders) deployment
Id1_SILVANUS platform should provide simulation of fire ignition scenarios
0.00
2.00
4.00
6.00
8.00
10.00
12.00
A
B
C
CV

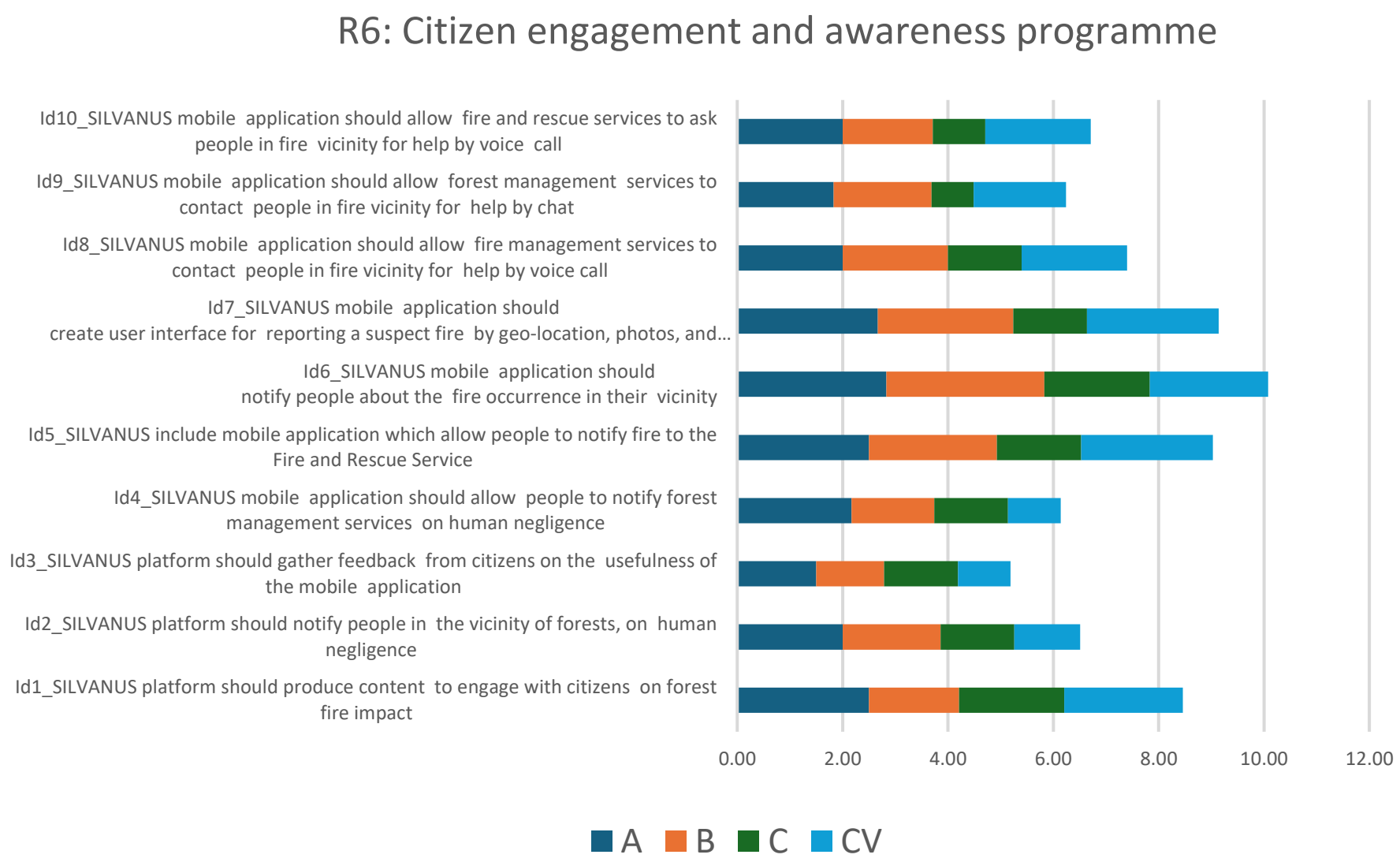
R6: Citizen engagement and awareness programme
Id10_SILVANUS mobile application should allow fire and rescue services to ask people in fire vicinity for help by voice call
Id9_SILVANUS mobile application should allow forest management services to contact people in fire vicinity for help by chat
Id8_SILVANUS mobile application should allow fire management services to contact people in fire vicinity for help by voice call
Id7_SILVANUS mobile application should create user interface for reporting a suspect fire by geo-location, photos, and...
Id6_SILVANUS mobile application should notify people about the fire occurrence in their vicinity
Id5_SILVANUS include mobile application which allow people to notify fire to the Fire and Rescue Service
Id4_SILVANUS mobile application should allow people to notify forest management services on human negligence
Id3_SILVANUS platform should gather feedback from citizens on the usefulness of the mobile application
Id2_SILVANUS platform should notify people in the vicinity of forests, on human negligence
Id1_SILVANUS platform should produce content to engage with citizens on forest fire impact
0.00
2.00
4.00
6.00
8.00
10.00
12.00
A
B
C
CV

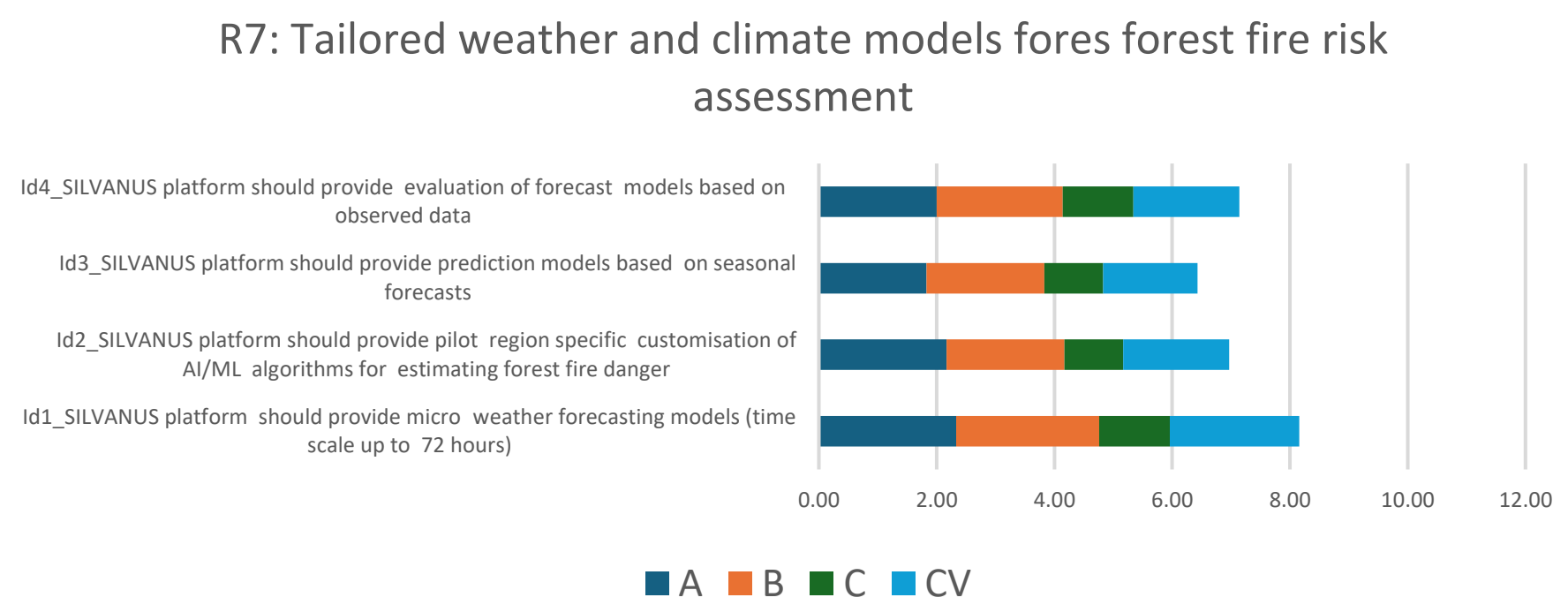
R7: Tailored weather and climate models fores forest fire risk assessment
Id4_SILVANUS platform should provide evaluation of forecast models based on observed data
Id3_SILVANUS platform should provide prediction models based on seasonal forecasts
Id2_SILVANUS platform should provide pilot region specific customisation of AI/ML algorithms for estimating forest fire danger
Id1_SILVANUS platform should provide micro weather forecasting models (time scale up to 72 hours)
0.00
2.00
4.00
6.00
8.00
10.00
12.00
A
B
C
CV

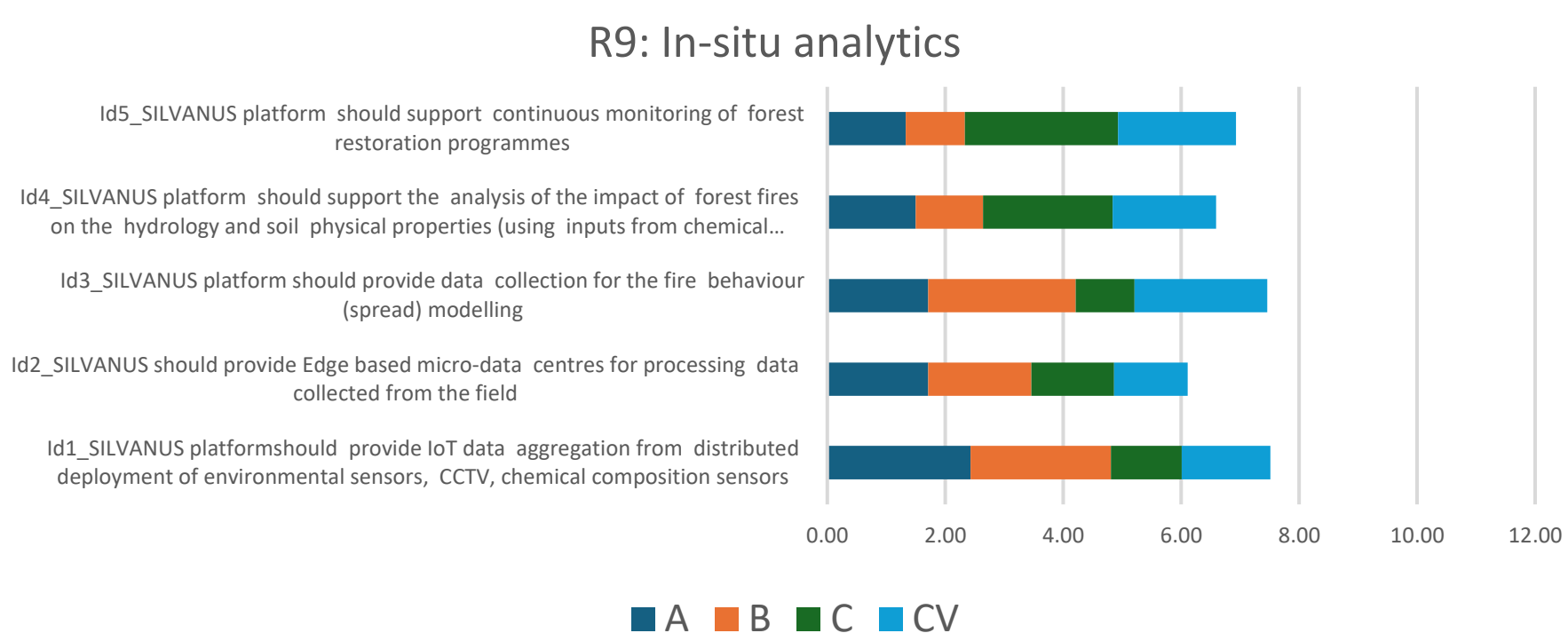
R9: In-situ analytics
Id5_SILVANUS platform should support continuous monitoring of forest restoration programmes
Id4_SILVANUS platform should support the analysis of the impact of forest fires on the hydrology and soil physical properties (using inputs from chemical...
Id3_SILVANUS platform should provide data collection for the fire behaviour (spread) modelling
Id2_SILVANUS should provide Edge based micro-data centres for processing data collected from the field
Id1_SILVANUS platformshould provide IoT data aggregation from distributed deployment of environmental sensors, CCTV, chemical composition sensors
0.00
2.00
4.00
6.00
8.00
10.00
12.00
A
B
C
CV

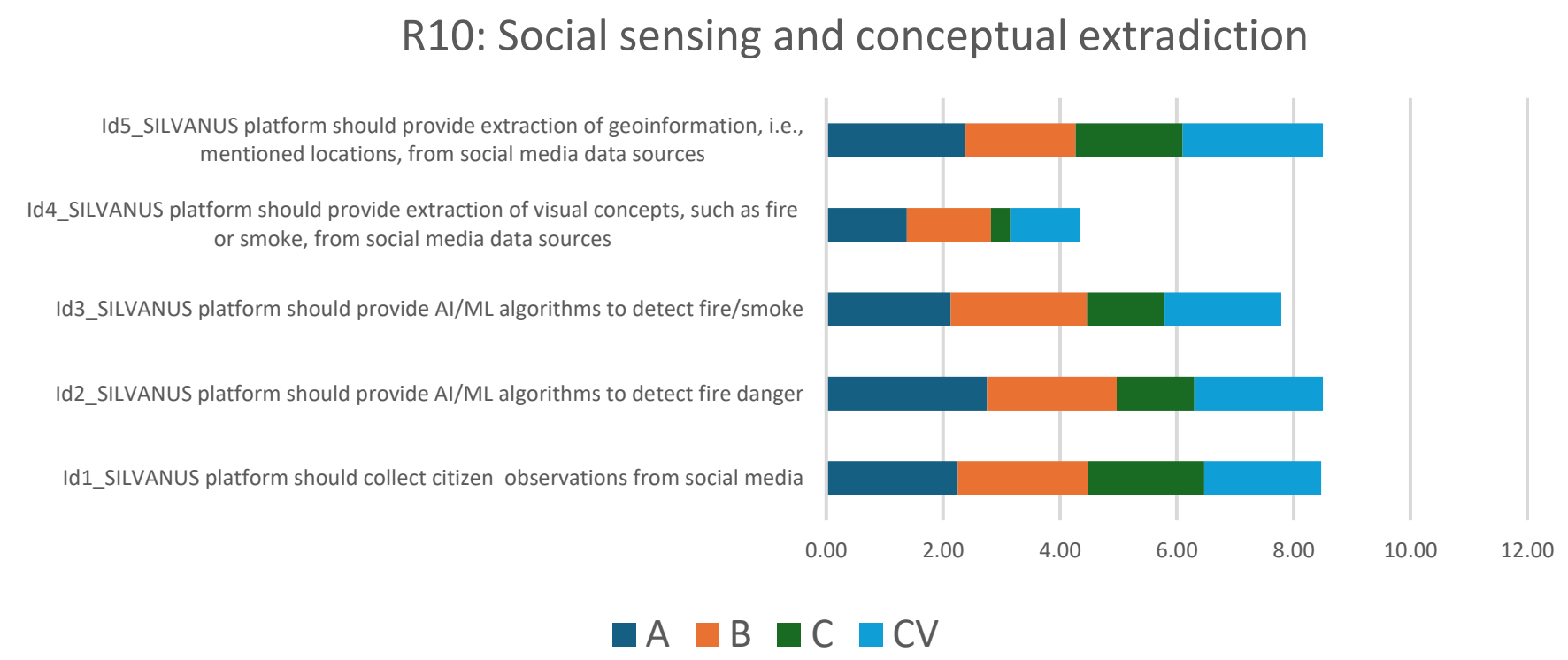
R10: Social sensing and conceptual extradiction
Id5_SILVANUS platform should provide extraction of geoinformation, i.e., mentioned locations, from social media data sources
Id4_SILVANUS platform should provide extraction of visual concepts, such as fire or smoke, from social media data sources
Id3_SILVANUS platform should provide AI/ML algorithms to detect fire/smoke
Id2_SILVANUS platform should provide AI/ML algorithms to detect fire danger
Id1_SILVANUS platform should collect citizen observations from social media
0.00
2.00
4.00
6.00
8.00
10.00
12.00
A
B
C
CV

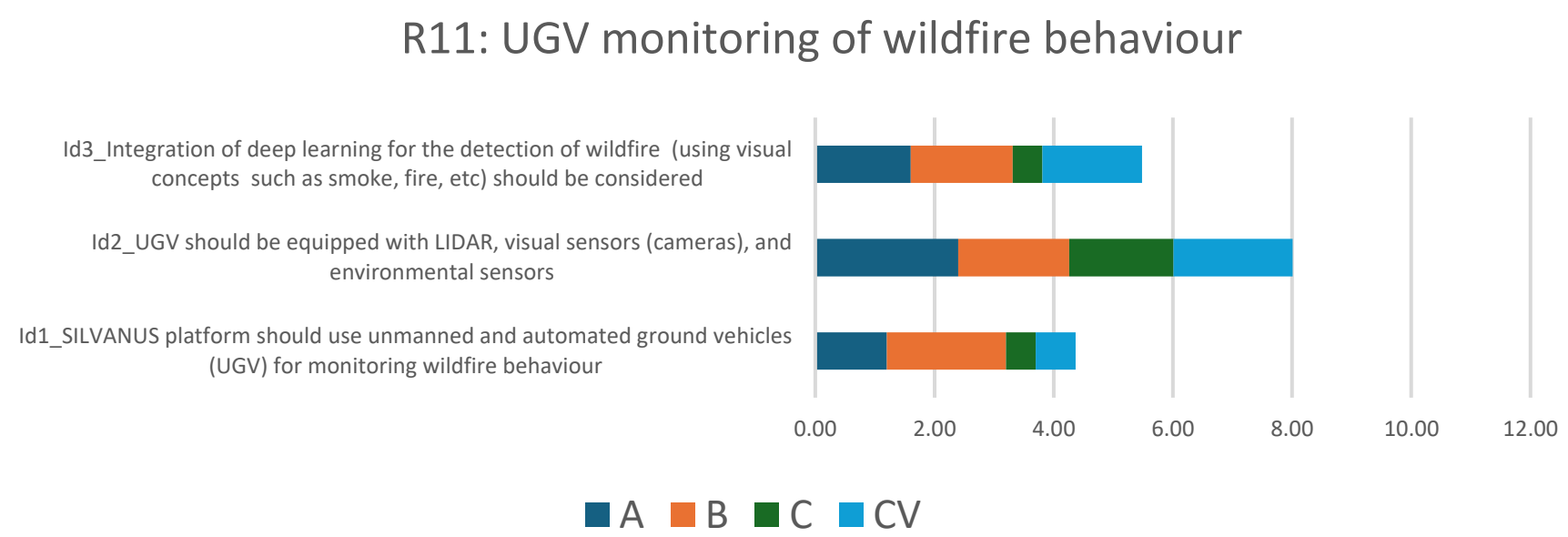
R11: UGV monitoring of wildfire behaviour
Id3_Integration of deep learning for the detection of wildfire (using visual concepts such as smoke, fire, etc) should be considered
Id2_UGV should be equipped with LIDAR, visual sensors (cameras), and environmental sensors
Id1_SILVANUS platform should use unmanned and automated ground vehicles (UGV) for monitoring wildfire behaviour
0.00
2.00
4.00
6.00
8.00
10.00
12.00
A
B
C
CV

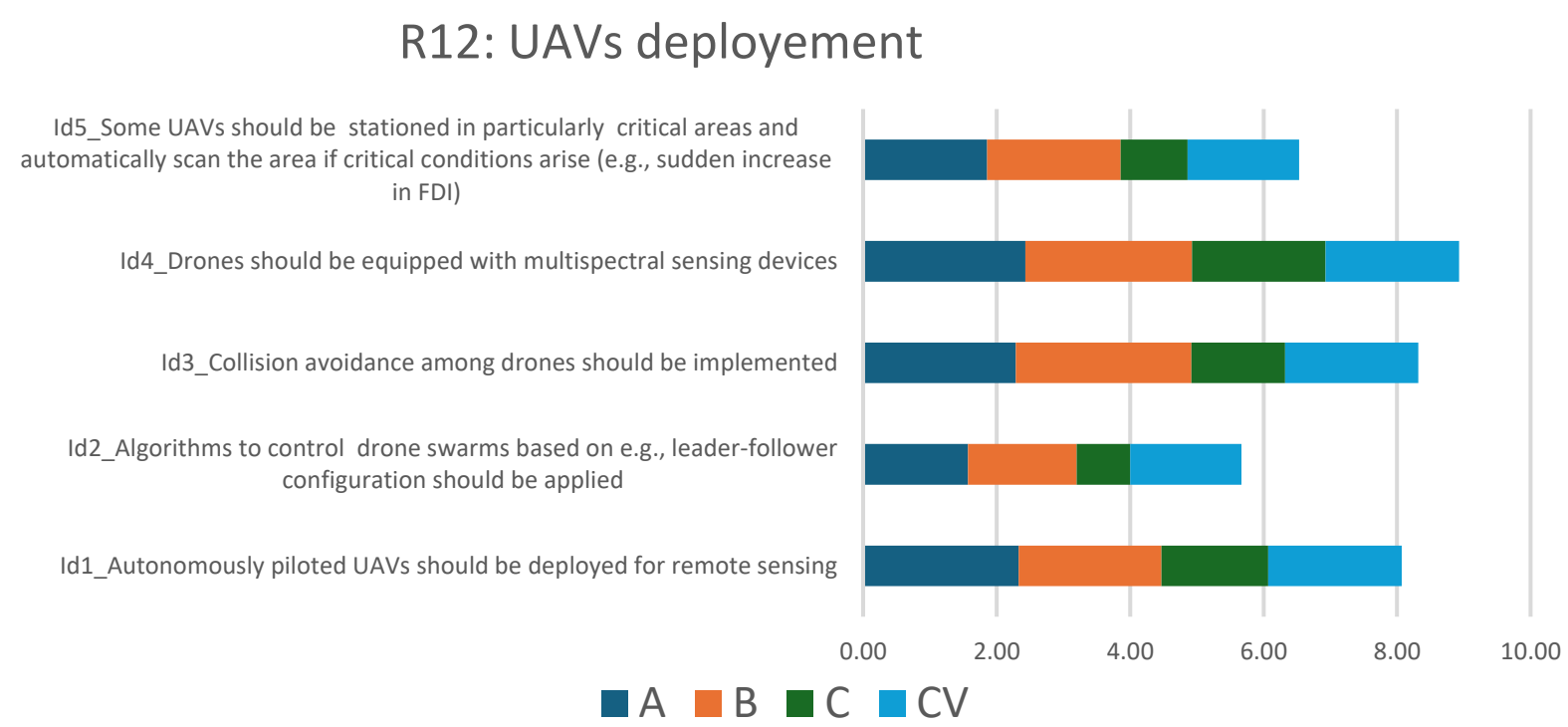
R12: UAVs deployement
Id5_Some UAVs should be stationed in particularly critical areas and automatically scan the area if critical conditions arise (e.g., sudden increase in FDI)
Id4_Drones should be equipped with multispectral sensing devices
Id3_Collision avoidance among drones should be implemented
Id2_Algorithms to control drone swarms based on e.g., leader-follower configuration should be applied
Id1_Autonomously piloted UAVs should be deployed for remote sensing
0.00
2.00
4.00
6.00
8.00
10.00
A
B
C
CV

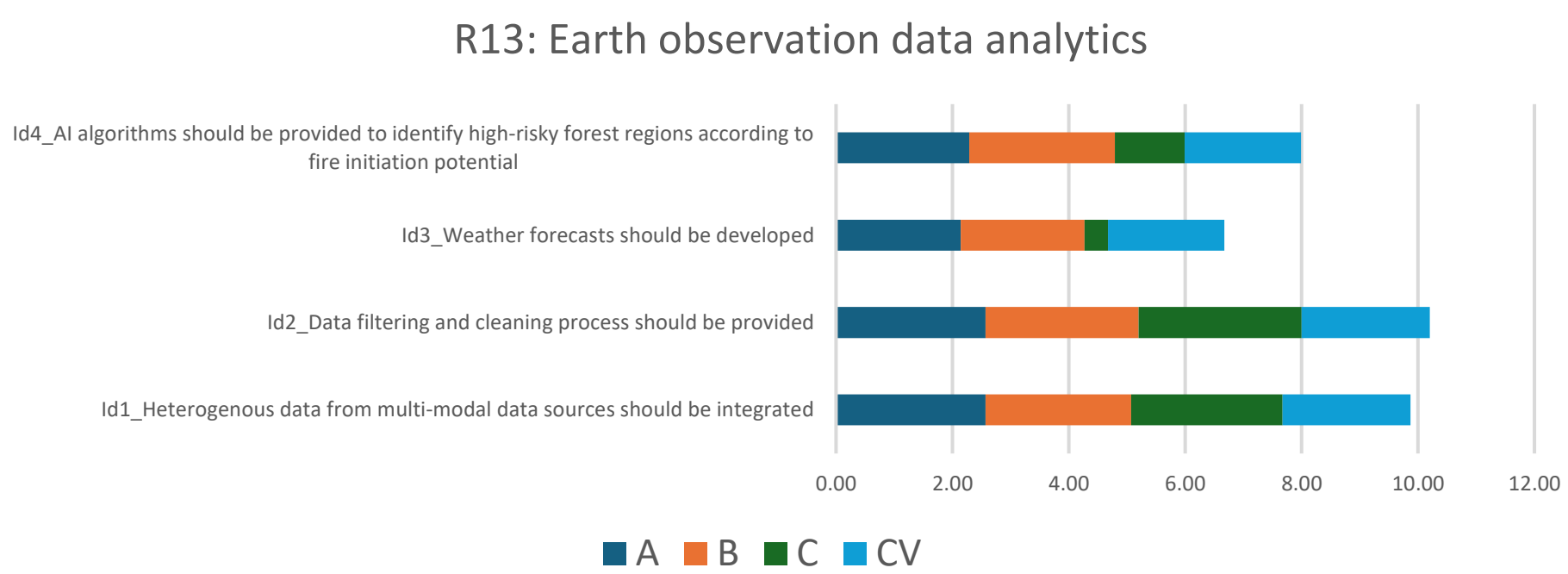
R13: Earth observation data analytics
Id4_AI algorithms should be provided to identify high-risky forest regions according to fire initiation potential
Id3_Weather forecasts should be developed
Id2_Data filtering and cleaning process should be provided
Id1_Heterogenous data from multi-modal data sources should be integrated
0.00
2.00
4.00
6.00
8.00
10.00
12.00
A
B
C
CV

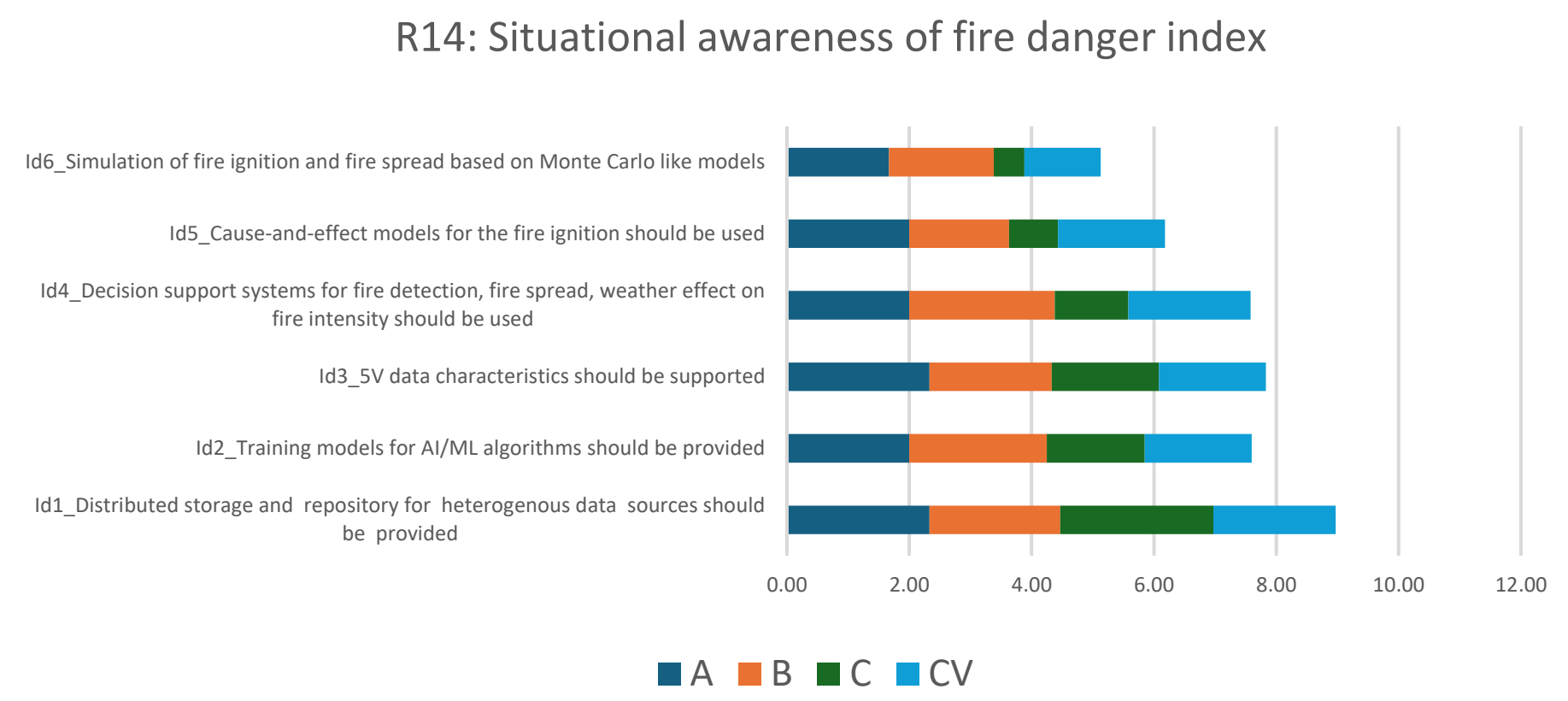
R14: Situational awareness of fire danger index
Id6_Simulation of fire ignition and fire spread based on Monte Carlo like models
Id5_Cause-and-effect models for the fire ignition should be used
Id4_Decision support systems for fire detection, fire spread, weather effect on fire intensity should be used
Id3_5V data characteristics should be supported
Id2_Training models for AI/ML algorithms should be provided
Id1_Distributed storage and repository for heterogenous data sources should be provided
0.00
2.00
4.00
6.00
8.00
10.00
12.00
A
B
C
CV

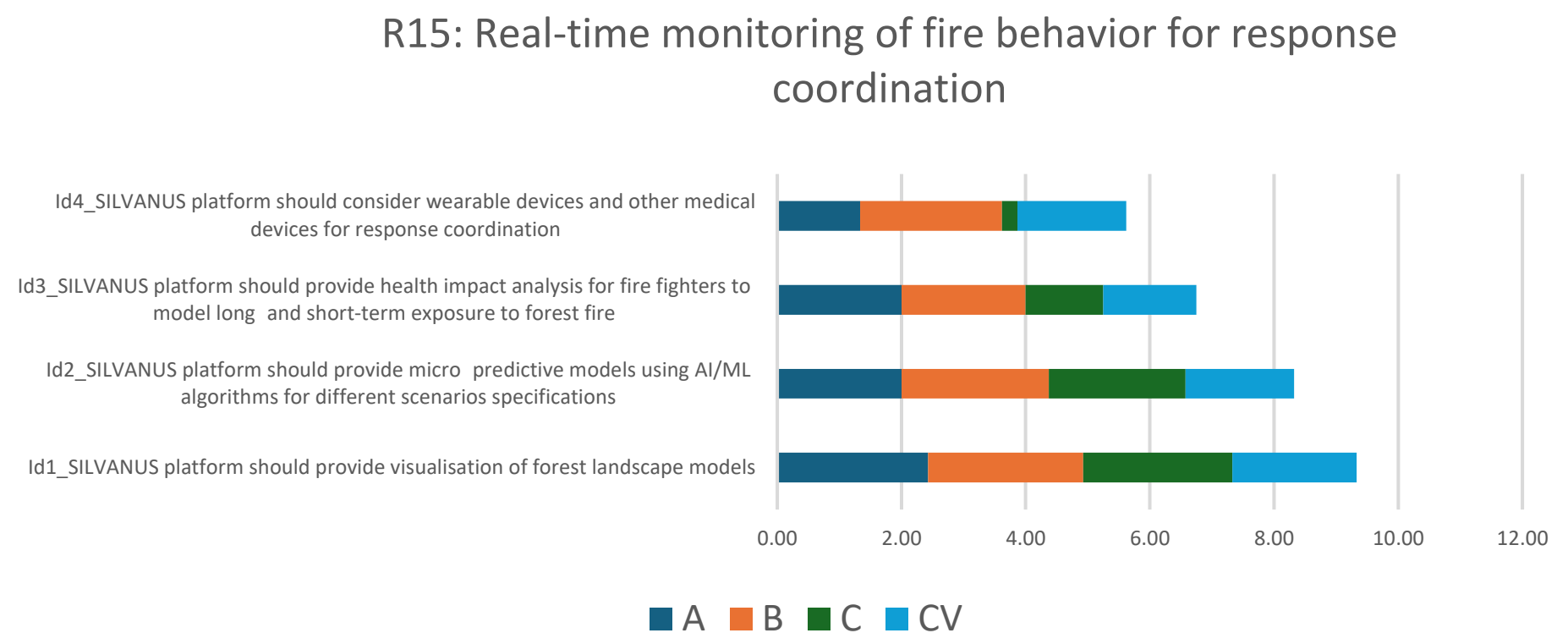
R15: Real-time monitoring of fire behavior for response coordination
Id4_SILVANUS platform should consider wearable devices and other medical devices for response coordination
Id3_SILVANUS platform should provide health impact analysis for fire fighters to model long and short-term exposure to forest fire
Id2_SILVANUS platform should provide micro predictive models using AI/ML algorithms for different scenarios specifications
Id1_SILVANUS platform should provide visualisation of forest landscape models
0.00
2.00
4.00
6.00
8.00
10.00
12.00
A
B
C
CV

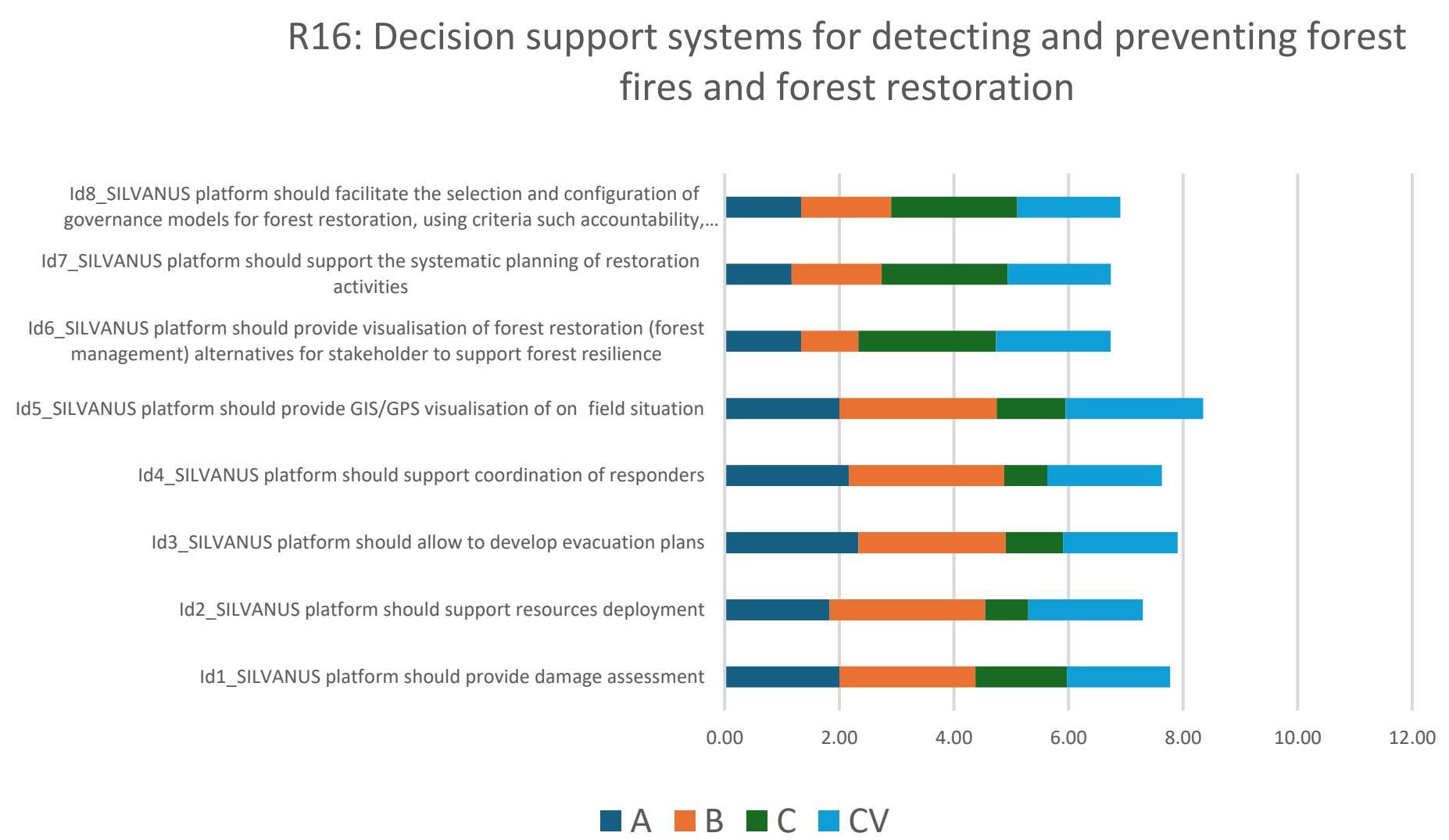
R16: Decision support systems for detecting and preventing forest fires and forest restoration
Id8_SILVANUS platform should facilitate the selection and configuration of governance models for forest restoration, using criteria such accountability,...
Id7_SILVANUS platform should support the systematic planning of restoration activities
Id6_SILVANUS platform should provide visualisation of forest restoration (forest management) alternatives for stakeholder to support forest resilience
Id5_SILVANUS platform should provide GIS/GPS visualisation of on field situation
Id4_SILVANUS platform should support coordination of responders
Id3_SILVANUS platform should allow to develop evacuation plans
Id2_SILVANUS platform should support resources deployment
Id1_SILVANUS platform should provide damage assessment
0.00
2.00
4.00
6.00
8.00
10.00
12.00
A
B
C
CV
